# Sentiment classification optimization based on improved Senti-BERT and BiLSTM-Attention

**DOI:** 10.1371/journal.pone.0358103

**Published:** 2026-09-15

**Authors:** Jie Zhang, Jianbang Liu, Zhaosheng Xu

**Affiliations:** 1 Faculty of Mathematics and Computer, Xinyu University, Xinyu, China; 2 Faculty of Computer and Mathematical Sciences, Universiti Teknologi MARA, Shah Alam, Malaysia; Philadelphia University, JORDAN

## Abstract

This study focuses on the problem of insufficient accuracy in sentiment classification in complex texts and multimodal scenarios. A sentiment classification optimization method is proposed, which integrates a bidirectional encoder representation Transformer model for sentiment analysis (Senti-BERT)and a bidirectional Long Short-Term Memory with Attention (BiLSTM-Attention). Firstly, the Senti-BERT layer is introduced to optimize the generation of word vectors by introducing an emotion dictionary leading mechanism, and a dynamic weighting strategy is adopted to fuse multi-modal features such as text, emoticons, and images. Subsequently, the hierarchical attention mechanism of BiLSTM-Attention structure is used to enhance contextual modeling. Finally, the stacking method is adopted to integrate the advantages of multiple models. The experimental results show that this method performs well in multiple dimensions. This optimization method achieved an F1 score of 84.6% in verb sentiment word recognition and 90.1% in adjective recognition, which is 4.8% higher than the bidirectional encoder representation Transformer. The full modal fusion results in a classification accuracy of 89.4%, and the English accuracy exceeds 90% in cross language tasks. The accuracy of emotion segment localization using dynamic weight attention mechanism reaches 90%. The quantization model maintains a low latency of 92ms under 10000QPS concurrency, and the Stacking strategy increases the AUC value to 0.943. The research provides a high-precision solution for social media sentiment analysis through innovative multi-level architecture design.

## 1. Introduction

With the rapid development of social media, user-generated content has grown explosively, containing rich emotional information that is of great value in applications such as public opinion monitoring, market analysis, and intelligent services. Sentiment analysis, as a key task in natural language processing, aims to automatically identify emotional tendencies from textual data and plays an important role in understanding user behavior and social dynamics [1]. However, with the increasing scale and complexity of data, traditional methods have gradually exposed limitations in feature representation and generalization performance in sentiment classification tasks [2]. Early sentiment analysis methods mainly relied on sentiment lexicons and rule-based approaches, which provided strong interpretability but struggled to adapt to dynamically changing language environments [3]. Subsequently, machine learning methods improved generalization through data-driven modeling, yet still depended heavily on manual feature engineering, making it difficult to fully capture complex semantic information. In recent years, deep learning approaches have significantly improved sentiment analysis performance through end-to-end modeling. In particular, methods based on recurrent neural networks and attention mechanisms have demonstrated strong capabilities in contextual modeling [4]. Furthermore, pre-trained language models have enhanced semantic representation by learning from large-scale corpora, showing strong adaptability in multilingual and low-resource scenarios and providing a new paradigm for sentiment analysis [5]. Despite these advancements, existing methods still face several challenges in handling complex texts and multimodal scenarios. On the one hand, current models have limitations in capturing long-range dependencies and fine-grained emotional expressions, making it difficult to fully model implicit contextual semantics. On the other hand, most studies focus on single-text modalities and lack effective integration of multimodal information such as emojis and images, which limits the ability to accurately reflect diverse emotional expressions [6,7]. In addition, the lack of explicit guidance for sentiment and opinion word identification further constrains model performance. To address these issues, this paper proposes an enhanced sentiment classification method that integrates an improved Senti-BERT with a BiLSTM-Attention framework. Specifically, a sentiment lexicon-guided mechanism is introduced to enhance semantic representation, while the BiLSTM-Attention structure is employed to strengthen contextual modeling and key sentiment feature extraction. Meanwhile, multimodal features, including text, emojis, and images, are jointly modeled to achieve cross-modal sentiment representation. Furthermore, a multi-model fusion strategy is adopted to improve the overall generalization ability and robustness of the model, thereby enhancing sentiment classification performance in complex scenarios.

## 2. Related work

With the continuous development of sentiment analysis, related methods have evolved from rule-based and traditional machine learning approaches to deep learning paradigms, and further extended toward multimodal modeling and explainable analysis. Different methods exhibit distinct advantages in terms of semantic representation capability, contextual modeling, and generalization performance.

In terms of traditional machine learning methods, X. Liu et al. proposed an improved multi-label k-nearest neighbor (L-MLkNN) algorithm to address the low accuracy and efficiency issues in short-text sentiment classification, achieving notable improvements in recall and classification performance [8]. Meanwhile, F. Liu et al. introduced a multimodal mixed emotion recognition framework, which incorporates channel attention mechanisms to effectively fuse multimodal features and handle the coexistence of positive and negative emotions [9]. In addition, J. L. Huan et al. enhanced text classification accuracy by integrating multidimensional document representations with recurrent neural networks [10], while Y. Bhanusree et al. developed a time-distributed attention-based convolutional neural network combined with a random forest classifier, reducing dependence on manual feature engineering and improving model stability [11]. However, such methods still show limitations in modeling complex semantic relationships.

With the advancement of deep learning, neural network-based approaches have become dominant. N. I. Ajali-Hernández et al. proposed a sentiment classification method combining attention mechanisms with long short-term memory networks, achieving high accuracy while maintaining low computational cost [12]. S. Zhang et al. developed a sentiment classification approach based on the ELECTRA pre-trained model, further enhanced by integrating a BiLSTM structure for improved performance on Chinese short-text data [13]. Furthermore, pre-trained language models significantly enhance semantic representation through self-supervised learning on large-scale corpora. C. Shaw et al. proposed a transfer learning-based IndoBERT model, demonstrating strong performance in Indonesian sentiment classification tasks [14]. T. Kumar et al. applied multilingual BERT to effectively recognize emotions in Hindi text [15], while A. Chriqui et al. developed the HeBERT model along with a dedicated sentiment analysis tool, improving fine-grained emotion recognition capability [16].

In recent years, sentiment analysis has further progressed toward complex structural modeling and explainability. On one hand, Graph Neural Networks (GNNs) have been introduced to capture intricate semantic dependencies by constructing relational graphs. H. Jiang et al. proposed the Fpa-GCN model, which enhances aspect sentiment triplet extraction through multi-branch graph convolution and gating mechanisms [17]. T. Meng et al. developed a heterogeneous graph-based multi-message passing framework to effectively model multimodal interactions in conversational emotion recognition tasks [18]. On the other hand, explainable sentiment analysis has gained increasing attention. T. M. A. U. Gunathilaka et al. proposed a fine-grained feature extraction approach based on BERT and CNN, improving interpretability through key sentence selection [19]. Z. Lin et al. introduced a multi-expert ensemble model that leverages multimodal sentiment annotation and multi-scale modeling, achieving improved performance in depression detection while enhancing model interpretability and robustness [20].

In summary, although significant progress has been achieved in sentiment classification, several challenges remain. Most existing methods focus on single-modality modeling and lack effective multimodal fusion capabilities, which limits their ability to capture complementary information across heterogeneous data sources. In addition, limitations persist in modeling complex semantic relationships and long-range dependencies, especially in short texts with sparse contextual information and implicit sentiment expressions. Furthermore, many deep learning models rely heavily on large-scale parameters, leading to high computational costs and reduced efficiency in real-world deployment scenarios. Meanwhile, the issue of model interpretability remains insufficiently addressed, as most methods fail to provide transparent explanations for their predictions, thereby reducing their reliability in sensitive application domains. Moreover, cross-domain generalization ability is still weak, and models often suffer from performance degradation when applied to different datasets or languages.

To address these issues, this study proposes a sentiment classification method that integrates an improved Senti-BERT with a BiLSTM-Attention framework, aiming to enhance sentiment recognition performance, contextual understanding, and generalization ability in complex scenarios while improving the balance between efficiency and interpretability.

## 3. Methods and materials

### 3.1 Senti-BERT model optimization for emotional enhancement

**Fig 1:**
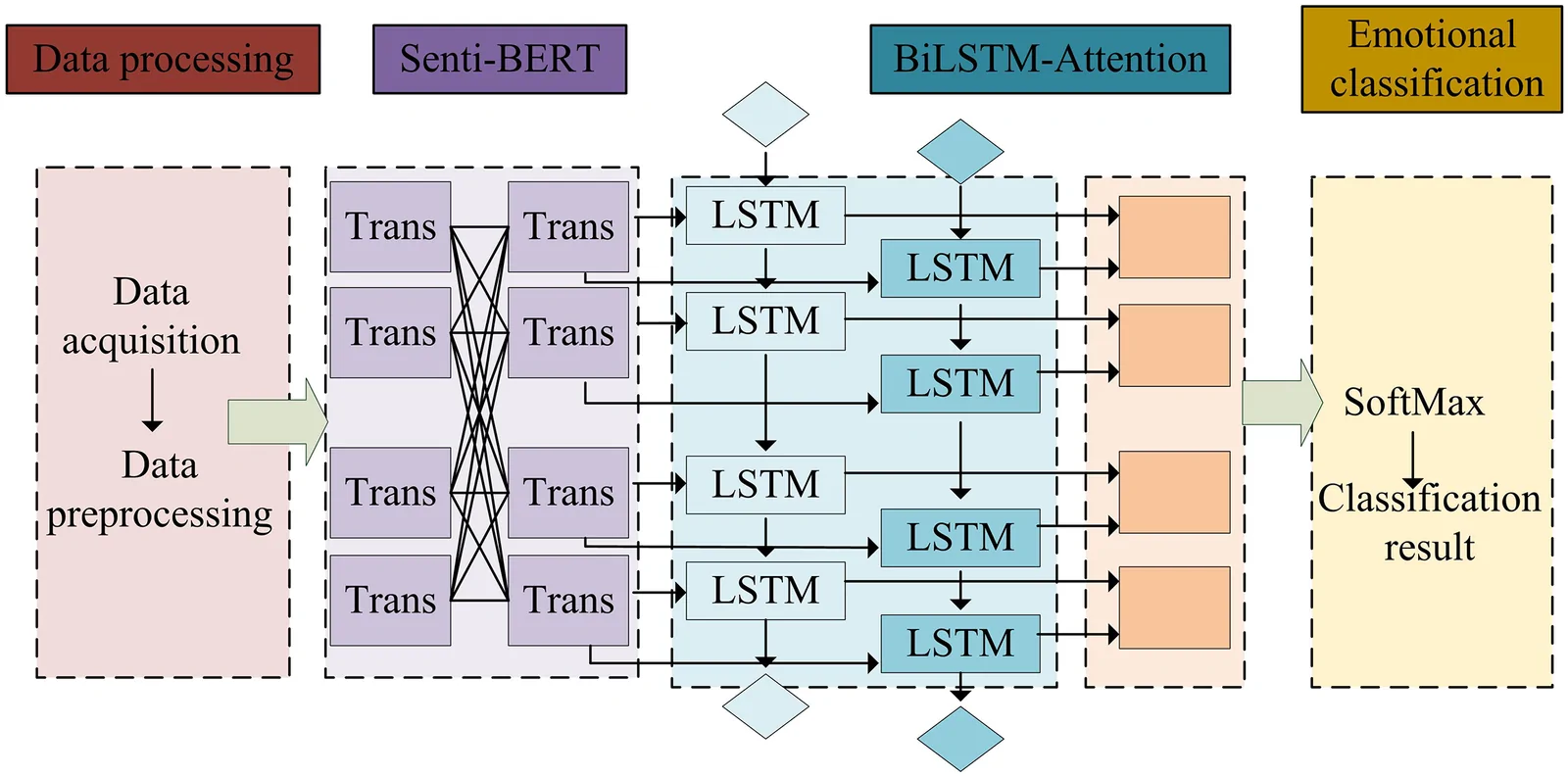
BERT-BiLSTM model frame diagram.

With the rapid development of social media, the Weibo platform has become a crucial arena for public opinion monitoring and analysis. The massive user base on this platform continuously shares real-time updates and opinions, generating multimodal data—— including text, images, and emojis that—— provide valuable insights into public sentiment. However, current sentiment analysis methods still face significant limitations: traditional approaches overly rely on single-text features while neglecting the critical role of multimodal information like images and emojis in emotional expression, resulting in inadequate understanding of complex semantics and contextual nuances. Moreover, existing technologies remain at the superficial level of sentiment polarity (positive, negative, neutral) judgment, lacking in-depth exploration and attribution analysis of emotional triggers, which severely restricts the practical value and interpretability of analytical results. To address these shortcomings, we innovatively propose a BERT-BiLSTM thematic sentiment analysis model that integrates multimodal features. By combining Senti-BERT’s deep semantic understanding module, BiLSTM-Attention temporal context feature extraction module, and multi-level semantic information fusion mechanism, the model effectively captures synergistic expressions of textual and visual information, establishing an end-to-end deep analysis framework as shown in Fig 1. This framework not only realizes the accurate identification of users’ emotional tendencies, but also further excavates the thematic inducements and semantic structures behind emotions, so as to complete the comprehensive analysis from superficial emotions to deep inducements, providing more scientific and comprehensive technical support for public opinion monitoring, user experience analysis and social emotion governance.

As shown in Fig 1, the proposed model consists of four main modules: the data processing module, the Senti-BERT feature extraction module, the BiLSTM-Attention contextual modeling module, and the emotional classification module. First, in the data processing stage, the raw text is preprocessed by removing noise using regular expressions (e.g., HTML tags and URLs), performing Chinese word segmentation, and filtering stop words, thereby improving data quality and providing standardized input for subsequent modeling [21,22]. Then, in the Senti-BERT module, the preprocessed text is transformed into vector representations using a pre-trained language model. Meanwhile, a sentiment lexicon-guided mechanism is introduced to enhance the encoding of sentiment words and opinion words, thereby strengthening the representation of emotional semantic features. This mechanism dynamically balances semantic and sentiment information to improve sentiment recognition accuracy [23,24]. Subsequently, the BiLSTM-Attention module further models the sequential features. The bidirectional long short-term memory network captures contextual dependencies in both forward and backward directions, while the attention mechanism assigns different weights to features at different time steps, highlighting key emotional information and generating more discriminative contextual representations [25]. Finally, in the emotional classification module, the fused feature vectors are fed into a Softmax classifier to predict the sentiment categories. Through multi-level feature extraction and fusion, the proposed framework achieves an end-to-end mapping from raw text to sentiment labels. The study also transformed emoji expressions and images into vector representations, as shown in equation (1). The formulation in equation (1) is based on the standard feature representation process of pretrained language models, convolutional feature extraction, and residual visual encoding. In this study, this general encoding framework is adapted to obtain unified vector representations of text, emoji, and image modalities [26].


$$\begin{array}{c} \left\{ \begin{array}{c} m=fBERT\left(M\right)\\ e=fCNN\left(E\right)\\ v=fResNet\;\left(I\right) \end{array}\right. \end{array}$$ (1)

In equation (1), m represents text features. e represents emoji expressions. v represents image features. fBERT is the text encoder. M is the input text. fCNN is the expression encoder, E is the expression image. fResNet is the visual encoder. I is the input image. Based on the above vector representations, the image feature extraction and fusion process is further refined. Specifically, the input images are first resized to a unified scale, and a pre-trained convolutional neural network is employed to extract high-level semantic features. These visual features are then projected into the same representation space as the textual features through a linear mapping, enabling cross-modal alignment. In terms of fusion position, multimodal features are not simply concatenated at the output layer. Instead, image and emoticon features are injected after the Senti-BERT generates textual embeddings and before the deep encoding stage, allowing them to participate in subsequent semantic modeling and enhancing contextual sentiment representation. During this process, the aligned visual and emoticon features are combined with textual embeddings in an intermediate representation layer, so that they can be jointly optimized with the contextual encoding procedure rather than being treated as independent inputs. For weight allocation, a dynamic weighting strategy is adopted, where the model automatically adjusts the contribution of each modality according to its importance in different samples during training. Specifically, the model learns to assign adaptive importance to textual, visual, and emoticon features by capturing their contextual relevance within each input instance. This mechanism is implemented through a learnable gating-like structure, which enables the model to selectively emphasize informative modalities while suppressing less relevant ones. As a result, the injected image features can be dynamically enhanced when they are semantically consistent with the text, or attenuated when they introduce noise or ambiguity. This mechanism avoids the limitations of fixed weights and improves the effectiveness and robustness of multimodal fusion.

Afterwards, it is fused with text features to enrich the emotional feature representation of the text, enabling the model to capture emotional information more comprehensively. The calculation is shown in equation (2). The fusion form in equation (2) follows the common weighted multimodal fusion strategy. Different from fixed-weight concatenation, the weights in this study are learnable parameters, which allow the model to dynamically adjust the contribution of text, emoji, and image features during training [27].


$$\begin{array}{c} h=\alpha t+\beta e+\gamma v \end{array}$$ (2)

In equation (2), h represents the fused multi-modal emotional features. α, β, and γ are learnable weights. It should be noted that the multimodal fusion weights are not manually assigned but are automatically learned during training. This mechanism can be regarded as an attention-based feature selection process, which reduces the risk of overfitting to a specific dataset. Moreover, the learned weight distributions remain relatively stable across different training runs and data splits, indicating robustness to weight variations. After the multi-modal feature fusion in equation (2), the model is optimized end-to-end through a composite loss function, and the total loss function $L_{total}$ is defined in equation (3). The loss function in equation (3) is designed according to the multi-objective optimization idea commonly used in deep learning. The classification loss is used as the main task objective, while the dictionary-guided loss and regularization loss are introduced as auxiliary constraints to enhance sentiment representation and reduce overfitting [28].


$$\begin{array}{c} L_{total}=\varepsilon L_{cls}+\mu L_{lex}+\lambda L_{reg}\; \end{array}$$ (3)

In equation (3), $L_{cls}$ represents the classification loss. $L_{lex}$ indicates dictionary guidance loss. $L_{reg}\;$ represents regularization loss. ε represents the weight of the main task, with a value of 0.7. μ represents the weight of the dictionary task, with a value of 0.2. λ represents the regularization coefficient, with a value of 0.1.

### 3.2 Improvement of BiLSTM-Attention structure

**Fig 2:**
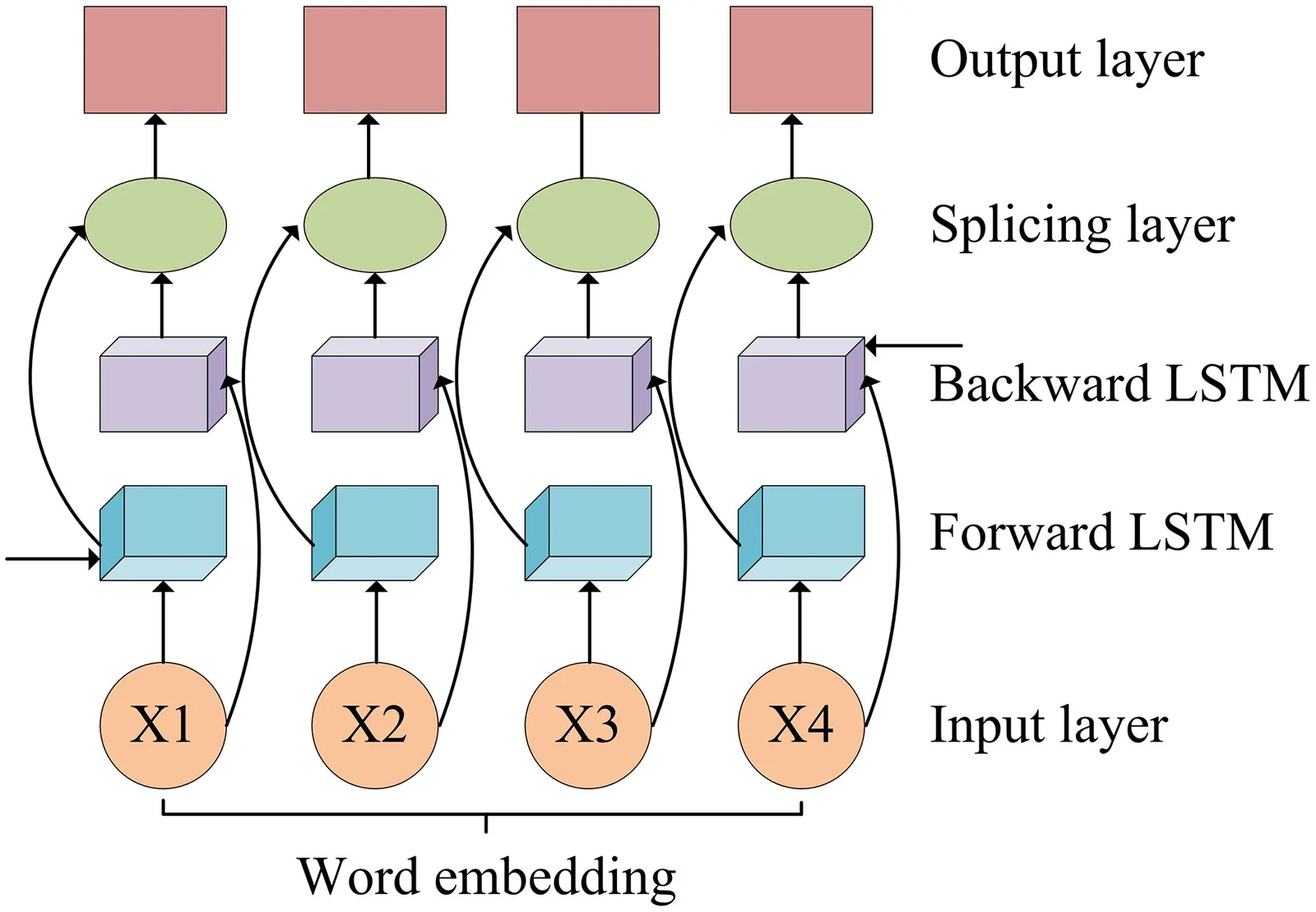
BiLSTM model framework.

In sentiment analysis tasks, the Senti-BERT layer effectively extracts local fine-grained emotional features by integrating an emotion dictionary with multimodal feature fusion mechanisms. However, traditional transformer models exhibit limitations in handling long-range emotional dependencies when processing long-span emotional expressions and complex contextual environments prevalent in social media texts. Conventional LSTM architectures struggle to capture deep interactions between emotional signals, while basic attention mechanisms often fail to precisely focus on critical emotional segments, resulting in incomplete and inconsistent grasp of global emotional semantics. To address these challenges, we propose an enhanced BiLSTM-Attention hierarchical architecture. This structure leverages BiLSTM to capture long-range temporal features and contextual information from text sequences, while introducing a hierarchical attentional weight allocation mechanism that improves model performance across three dimensions: 1) Establishing coherent modeling of cross-sentential emotional cues to enhance semantic integration of scattered emotional expressions; 2) Optimizing semantic relationship analysis between emotional words to improve inference quality of implicit emotional logic; 3) Achieving dynamic integration and interactive response between text and multimodal features like images and emojis. This architecture significantly enhances the model’s ability to comprehend global emotional semantics, providing an effective solution for deep emotional understanding in complex social media environments. The specific architecture of the BiLSTM-Attention model is illustrated in Fig 2.

Fig 2 shows the hierarchical architecture of BiLSTM, whose input features are derived from the output sequence (x_1_, x_2_, x_3_, x_4_,..., x_n_) of the Senti-BERT layer. This structure achieves context modeling through bidirectional processing units that operate in parallel: forward LSTM extracts historical contextual features along time series, and reverse LSTM captures subsequent contextual information in reverse. Finally, the bidirectional representations are fused into a comprehensive vector through feature concatenation operation, and its mathematical expression is shown in equation (4). The bidirectional modeling process in equation (4) is based on the standard BiLSTM sequence representation structure. In this study, the forward and backward hidden states are further integrated through a dynamic weighting parameter to strengthen contextual sentiment representation [29].


$$\begin{array}{c} \left\{ \begin{array}{c} \vec{F}=LSTM_{f}\left({h_{\text{1}}}^{\left(f\right)},{h_{\text{2}}}^{\left(f\right)},...,{h_{T}}^{\left(f\right)}\right)\\ \overset{\leftarrow }{B}=LSTM_{b}\left({h_{\text{1}}}^{\left(b\right)},{h_{\text{2}}}^{\left(b\right)},...,{h_{N}}^{\left(b\right)}\right)\\ H=\omega \vec{F}+(1-\omega )\overset{\leftarrow }{B} \end{array}\right. \end{array}$$ (4)

**Fig 3:**
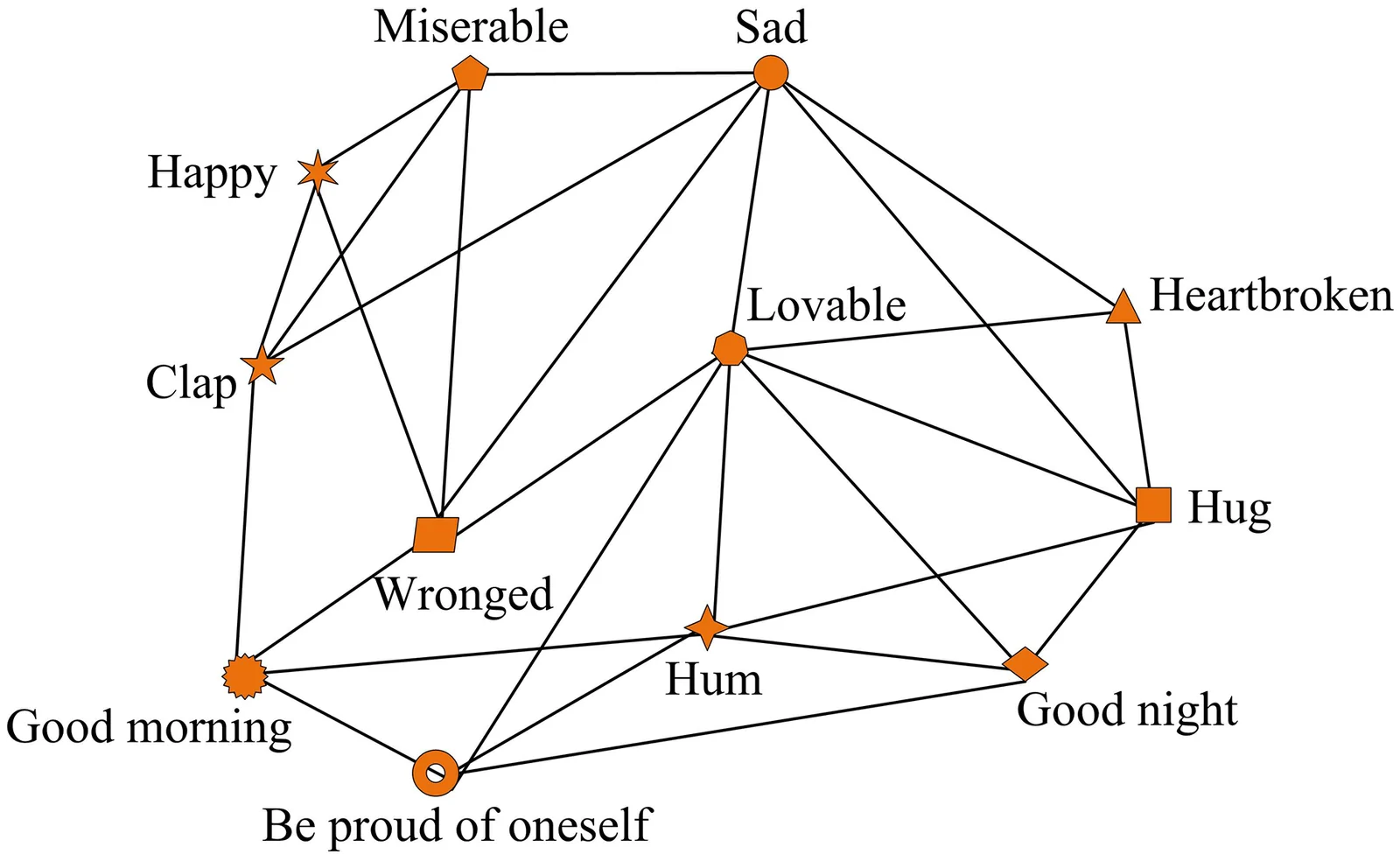
Emoji symbol network diagram.

In equation (4), $\vec{F}$ is the final state vector of the forward LSTM. $LSTM_{f}$ is the LSTM unit for forward propagation. ${h_{k}}^{\left(f\right)}$ is the hidden state of the forward propagation atstep k. $\overset{\leftarrow }{B}$ is the final state vector of the reverse LSTM. $LSTM_{b}$ is the LSTM unit for backpropagation. ${h_{m}}^{\left(b\right)}$ is the hidden state for step m of backpropagation. H represents the integrated features after fusion. ω is the dynamic weight parameter, with the range of [0,1]. Through in-depth analysis of the Sina Weibo dataset, the research found that existing sentiment analysis methods have significant shortcomings in processing emojis. Traditional models often treat emojis as static sentiment labels and ignore their semantic changes in different contexts. To capture the semantic relationships among emojis, this study constructs an emoji relationship network based on the co-occurrence characteristics of high-frequency emojis in the dataset. Specifically, the co-occurrence frequency of different emojis within the same text is first calculated, and their contextual representations are used to compute cosine similarity as a measure of semantic association strength. Subsequently, emojis are treated as nodes and similarity values as edge weights to build a topological structure, which is then visualized as the network shown in Fig 3.

In Fig 3, 12 high-frequency emoji expressions exhibit significant emotional expression differences in a diverse text environment, and a single emotional annotation is difficult to accurately reflect their true semantics. To overcome this limitation, a three-stage innovative plan is proposed: firstly, an emoji network is constructed based on massive Weibo data, and the semantic correlation between emoticons is quantified through statistical analysis methods. The node weights in this network reflect the frequency of expression usage, while the edge weights represent the co-occurrence probability of expressions, as expressed mathematically in equation (5). The similarity measure defined in equation (5) is derived from co-occurrence statistics and is conceptually related to cosine-normalized association measurement. In this study, it is adapted to evaluate the semantic association strength between emojis by normalizing their co-occurrence frequency with their independent occurrence frequencies [30].


$$\begin{array}{c} sim\left(e_{i},e_{j}\right)=\frac{freq\left(e_{i}\cap e_{j}\right)}{\sqrt{freq\left(e_{i}\right)\times freq\left(e_{j}\right)}} \end{array}$$ (5)

In equation (5), $sim\left(e_{i},e_{j}\right)$ is the similarity between emojis $e_{i}$ and $e_{j}$. $freq\left(e_{i}\cap e_{j}\right)$ is the co-occurrence frequency of $e_{i}$ and $e_{j}$ in the data. $freq\left(e_{i}\right)$ is the frequency of independent appearance of $e_{i}$. $freq\left(e_{j}\right)$ is the frequency of independent appearance of $e_{j}$. The similarity measure defined in equation (5) is derived from co-occurrence statistics and is conceptually related to cosine similarity. Specifically, it normalizes the co-occurrence frequency of emoji pairs by their individual occurrence frequencies, enabling a more balanced representation of their semantic association. Secondly, a hierarchical adaptive attention architecture is designed to address the issue of insufficient processing of emoticons by traditional attention mechanisms. This mechanism dynamically calculates the expression weight of each emoji by capturing contextual features through bidirectional LSTM. It should be noted that the hierarchical attention mechanism adopted in this study does not follow the conventional word-level and sentence-level structure. Instead, it consists of a time-step attention over BiLSTM outputs and a multimodal attention layer for integrating textual, emoticon, and image features, enabling multi-level sentiment representation. Its calculation is shown in equation (6). The attention weight calculation in equation (6) is based on the general neural attention mechanism. In this study, the attention score is calculated by jointly considering the BiLSTM hidden state and emoji feature at each time step, so that key emotional segments and context-dependent emoji semantics can be assigned higher weights [31].


$$\begin{array}{c} \theta_{t}\;=\frac{exp\left(W^{T}\left[h_{t}\;;e_{t}\;\right]\right)}{\sum_{k=1}^{T}\text{\textbackslash{}aa}exp\left(W^{T}\left[h_{k}\;;e_{k}\;\right]\right)}\; \end{array}$$ (6)

In equation (6), $\theta_{t}\;$ is the attention weight at the moment t. $W^{T}$ is a learnable weight matrix. $h_{t}$ is the hidden state of LSTM at time t. $e_{t}$ is the emoji feature at time t. T is the total time step of the sequence. k is a temporary iteration variable. Due to the inability of BiLSTM layers to distinguish the importance of text words in sentiment analysis, attention mechanisms were introduced to identify the semantic weights of words in order to improve classification accuracy. When combining BiLSTM and attention mechanism, the original softmax layer is replaced with an attention layer, and the softmax function is applied to convert the classification score into probability. In the semantic synthesis stage, text and emoji features are weighted and summed through self attention layers to calculate classification label scores. Then, the features are mapped to the target space through linear transformation to form the final feature representation for sentiment analysis. Its calculation is shown in equation (7). The nonlinear transformation in equation (7) follows the standard neural feature mapping form. It projects different input representations into a shared hidden space through learnable weight matrices and a nonlinear activation function, thereby enhancing the interaction between heterogeneous semantic features [32].


$$\begin{array}{c} D=tanh\left(Ws\cdot S+Wr\cdot R+b\right) \end{array}$$ (7)

In equation (7), Ws and Wr are the weight matrices. S and R are input vectors 1 and 2. b is a bias vector. In the sentiment analysis stage, the study uses the softmax function to perform sentiment classification on the text. Emotional tendencies are strictly classified into two categories: positive and negative. The specific calculation method and loss function L are shown in equation (8). The Softmax function and cross-entropy loss in equation (8) are standard classification formulations in neural network-based sentiment classification. They are used to convert sentiment scores into category probabilities and optimize the model parameters according to the ground-truth labels [33].


$$\begin{array}{c} \left\{ \begin{array}{c} p_{c}=\frac{exp\left(d_{c}\right)}{\sum_{r=1}^{c}exp\left(d_{r}\right)}\\ L=\sum_{d\in D}\sum_{c=1}^{c}p_{c}^{g}\left(d\right)\left(logp_{c}\left(d\right)\right) \end{array}\right. \end{array}$$ (8)

In equation (8), $p_{c}$ represents the predicted probability of category c. $d_{c}$ is the original score for category c. $d_{r}$ is the original score for category r. r is the index of categories. c is the total number of categories. d is input data. D is the training dataset. $p_{c}^{g}\left(d\right)$ is the true label of sample d. $p_{c}\left(d\right)$ is the predicted probability of sample d.

### 3.3 Enhancement of multi-model fusion strategy

**Fig 4:**
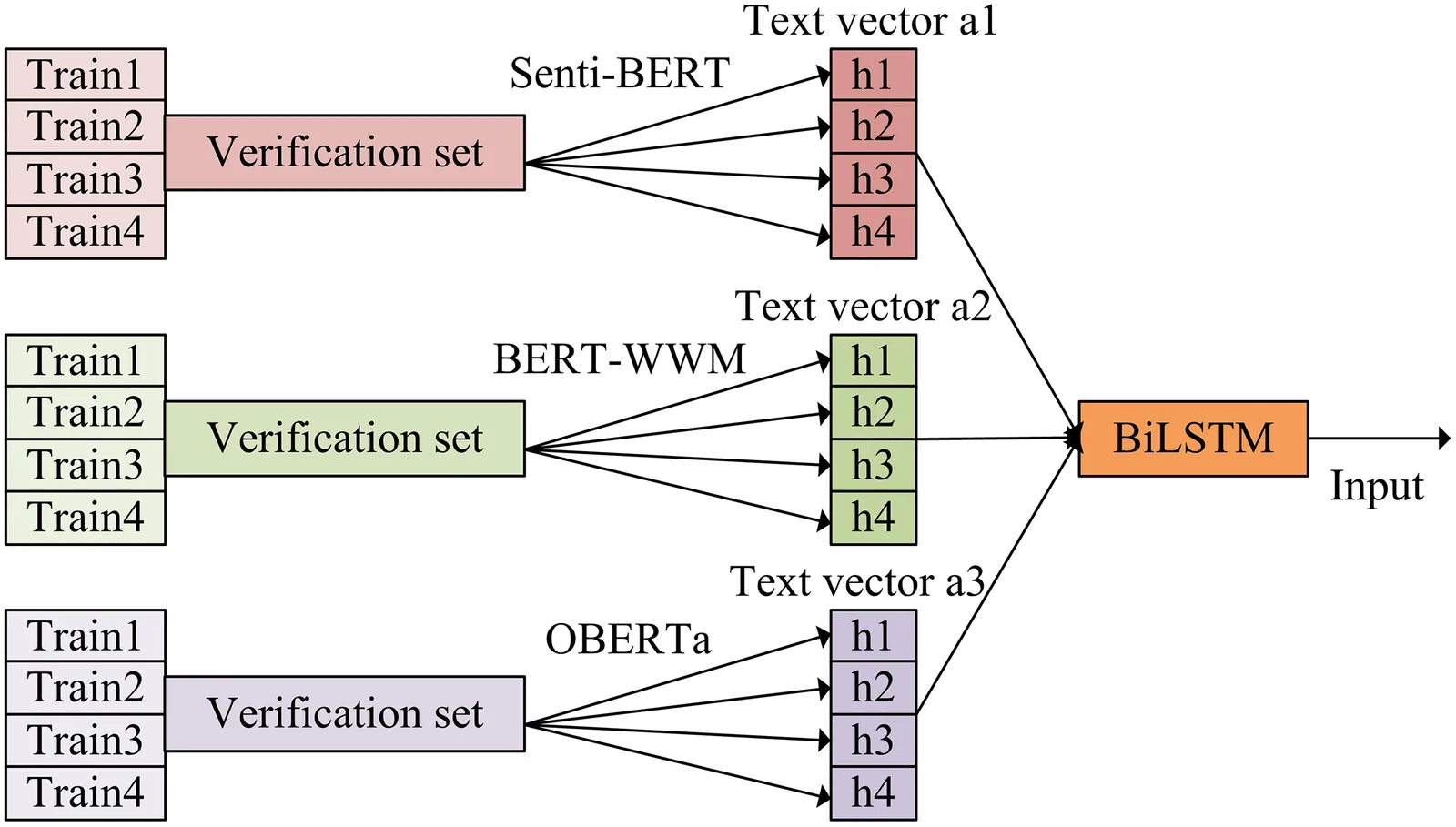
Multi-model fusion structure diagram.

After completing preliminary tasks such as data collection, cleaning, annotation, and multimodal text vectorization, sentiment analysis still faces critical challenges including insufficient model generalization capabilities and limited feature extraction dimensions. These issues severely constrain the accuracy and interpretability of models in real-world complex scenarios, limiting the depth of sentiment semantic mining. Single models often struggle to comprehensively capture diverse and implicit emotional patterns in social media texts, particularly showing significant performance degradation when encountering domain migration, stylistic variations, or cross-cultural contexts. To address these limitations, this study proposes a multi-model fusion integration approach that combines the strengths of different structural models to enhance overall sentiment analysis discriminative capabilities and stability. The core function of multi-model fusion lies in integrating prediction results from various base models through strategies like weighted voting, stacked generalization, or meta-learning, effectively reducing potential biases and overfitting risks associated with single models, thereby improving prediction accuracy and robustness. Additionally, this method enhances model adaptability to unknown data and cross-domain corpora by employing multi-perspective, multi-level feature representations and decision-making mechanisms, ensuring high performance even under distribution differences and boosting generalization capabilities. To achieve efficient collaboration and information complementarity among multiple models, the study further constructs a well-structured multi-model fusion framework, as illustrated in Fig 4.

**Fig 5:**
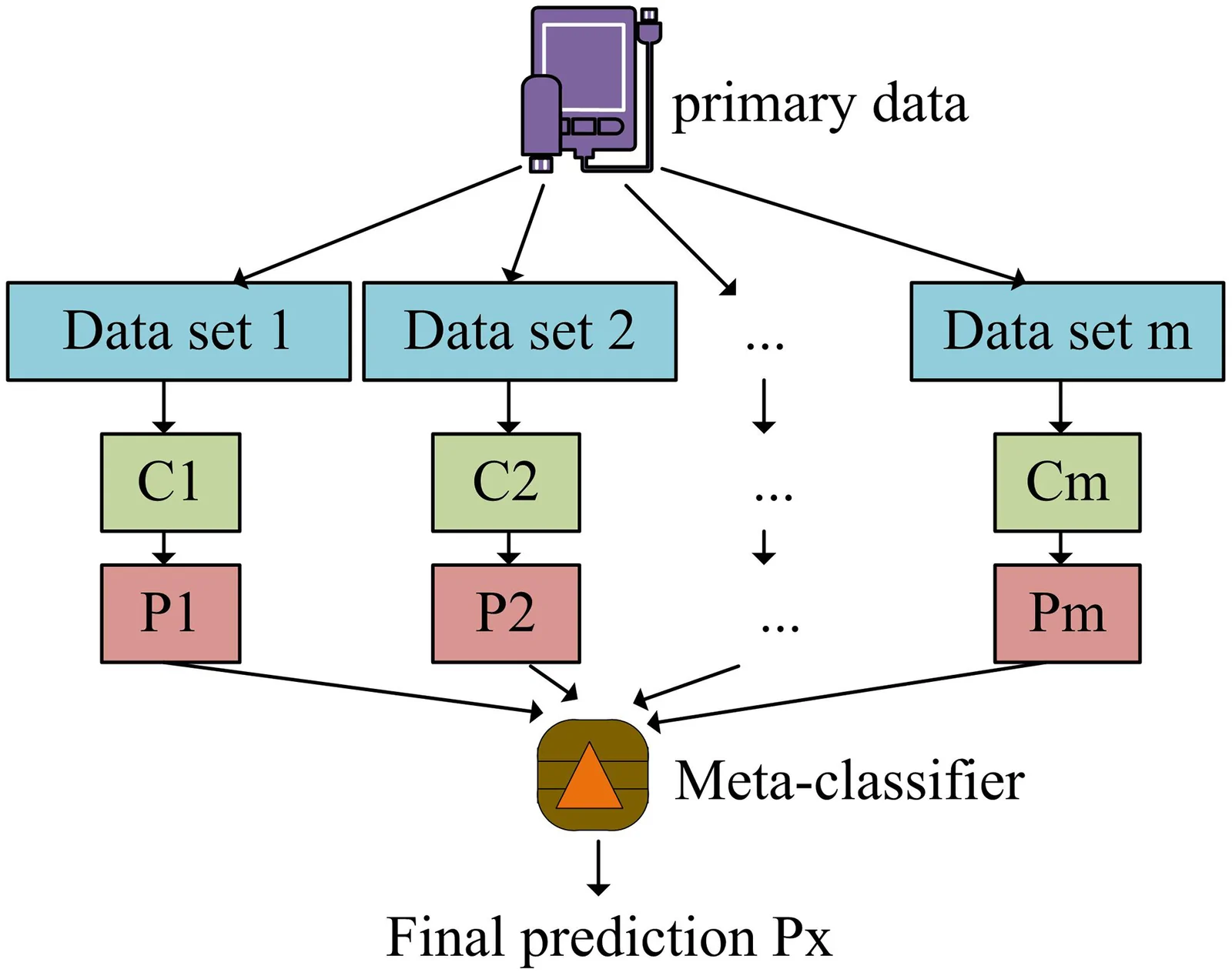
Flow chart of stacking algorithm.

Fig 4 shows that the dataset is divided into a training set and a validation set, which are processed by three pre trained models to generate text vectors. These vectors are input into the BiLSTM model and output sentiment classification results, demonstrating that model fusion improves the accuracy of sentiment analysis. Structural diagrams guide model fusion, promoting efficient feature learning and analysis. The study chooses the Stacked Generalization (Stacking) method to integrate the BERT model, as it integrates prediction results through layered training, improves performance, and reduces overfitting. Bootstrap Aggregating (Bagging) random sampling improves stability, but the prediction improvement is limited. Boosting gradually improves performance, which may lead to overfitting and complex training. Blending may not perform as well as Stacking in handling imbalanced datasets and complex tasks. The fusion of BERT models requires comprehensive advantages, balance stability and performance, and the Stacking algorithm is the best choice due to its hierarchical training and integration capabilities. The flowchart of the Stacking algorithm is shown in Fig 5.

Fig 5 shows the hierarchical processing flow of the Stacking algorithm. The raw data is processed in parallel by multiple sets of base classifiers to generate prediction results. These intermediate outputs are used as new feature inputs to the meta classifier for secondary learning, and the final output is ensemble prediction. In this study, logistic regression is adopted as the meta-learner to perform weighted fusion of the outputs from base models. This selection is grounded in the observation that the base learners (e.g., Senti-BERT and BiLSTM-Attention) already generate discriminative probabilistic predictions, such that the primary objective of the stacking stage is to recalibrate and integrate these outputs rather than to introduce additional nonlinear feature transformations. Logistic regression operates directly in the probability space, ensuring consistency in probabilistic interpretation while enabling effective aggregation of heterogeneous model outputs. Furthermore, considering that the meta-level training data in stacking are typically limited, the use of a low-capacity linear model helps mitigate the risk of overfitting and enhances generalization stability. Compared with simple averaging or voting strategies, logistic regression can adaptively learn the contribution of each base model based on its predictive reliability, thereby providing a more principled and flexible fusion mechanism. At the same time, in contrast to more complex meta-learners such as neural networks, it achieves a favorable balance between model complexity and integration effectiveness. This architecture combines the advantages of base model diversity and high-order feature combination through two-level modeling.

In summary, the study proposes an emotion classification optimization method that integrates improved Senti-BERT and BiLSTM-Attention. This method first vectorizes the text data through the Senti-BERT layer, then uses the BiLSTM-Attention model to extract contextual features, and strengthens the recognition of key emotional features through attention mechanisms. In addition, a multi model fusion strategy, especially the Stacking algorithm, is introduced to further enhance the model’s generalization ability and prediction accuracy. Through this multi-level and multimodal fusion method, the model can more effectively understand and analyze emotional information in complex texts, providing a new solution for emotional classification tasks.

## 4. Results

### 4.1 Performance verification of core algorithm components of sentiment analysis model

The study systematically evaluated the performance advantages of the proposed Senti-BERT and BiLSTM-Attention fusion model in sentiment classification tasks through experimental design. The experiments adopted a multi-dimensional assessment framework covering core metrics such as model accuracy, computational efficiency, and cross-scenario adaptability. Meanwhile, a statistical analysis of the dataset was conducted, showing that the proportions of positive, negative, and neutral samples are approximately 35%, 33%, and 32%, respectively, indicating a relatively balanced distribution. To ensure the reliability and reproducibility of experimental results, the research established a rigorous experimental infrastructure system, as detailed in Table 1. This system integrates the complete technical chain from hardware infrastructure to algorithm implementation, with particular emphasis on standardized management of environmental controls, data quality, and training processes. The experimental design fully considered the requirements for multimodal data processing and industrial deployment conditions in practical applications, comprehensively validating model performance through quantitative metrics and comparative analysis.

**Table 1:** Summary of experimental basic settings.

Configuration category	Parameter	Configuration
Hardware	GPU	NVIDIA V100/ A100/ T4
	Memory	32 GB DDR4
	Storage	NVMe SSD (1 TB)
Software	Python	3.8+
	Framework	PyTorch 1.12.0
	CUDA/cuDNN	CUDA 11.7/ cuDNN 8.5
Model	Senti-BERT	12 layers, 768 hidden size, 12 heads
	BiLSTM	2 layers, 512 units
	Attention	Dynamic attention (64-dim)
Training	Optimizer	AdamW (5e-5)
	Batch size	32
	Epochs	50
	Loss	Composite loss (main task weight 0.7)
Data	Dataset	50,000 Weibo samples
	Split	8:1:1
	Text length	512
	Image size	224 × 224
Reproducibility	Random seed	42
	Initialization	Xavier
	Early stopping	5 epochs

**Fig 6:**
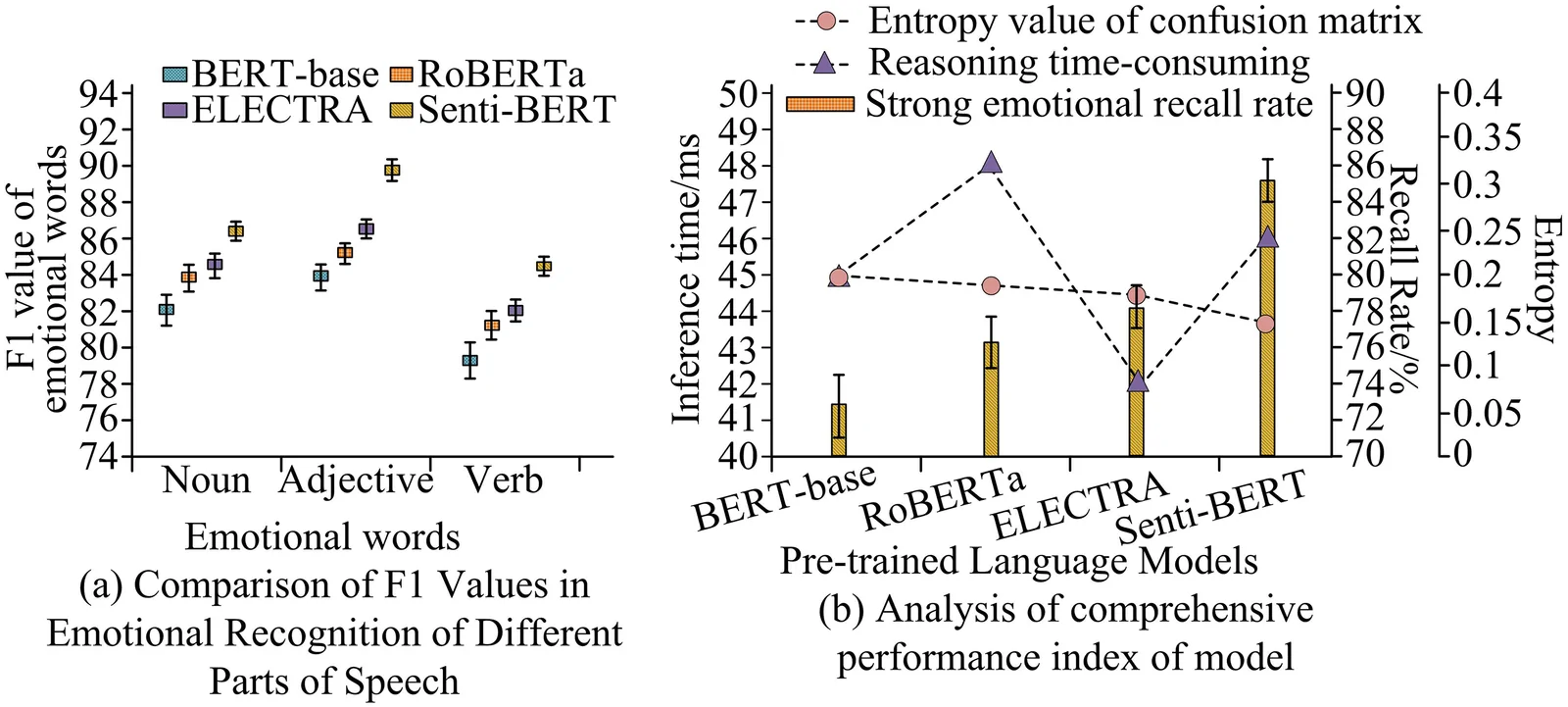
Multi-dimensional evaluation of affective analysis ability of pre-training language model.

To verify the optimization effect of Senti-BERT on sentiment word recognition tasks, a basic model comparison experiment was designed and studied. The comparative methods included the original BERT, Robust Optimized BERT Approach (RoBERTa), ELECTRA, and Senti-BERT. The evaluation index is mainly based on F1 value, supplemented by emotional word recall rate and confusion matrix analysis. The experimental results are shown in Fig 6.

**Fig 7:**
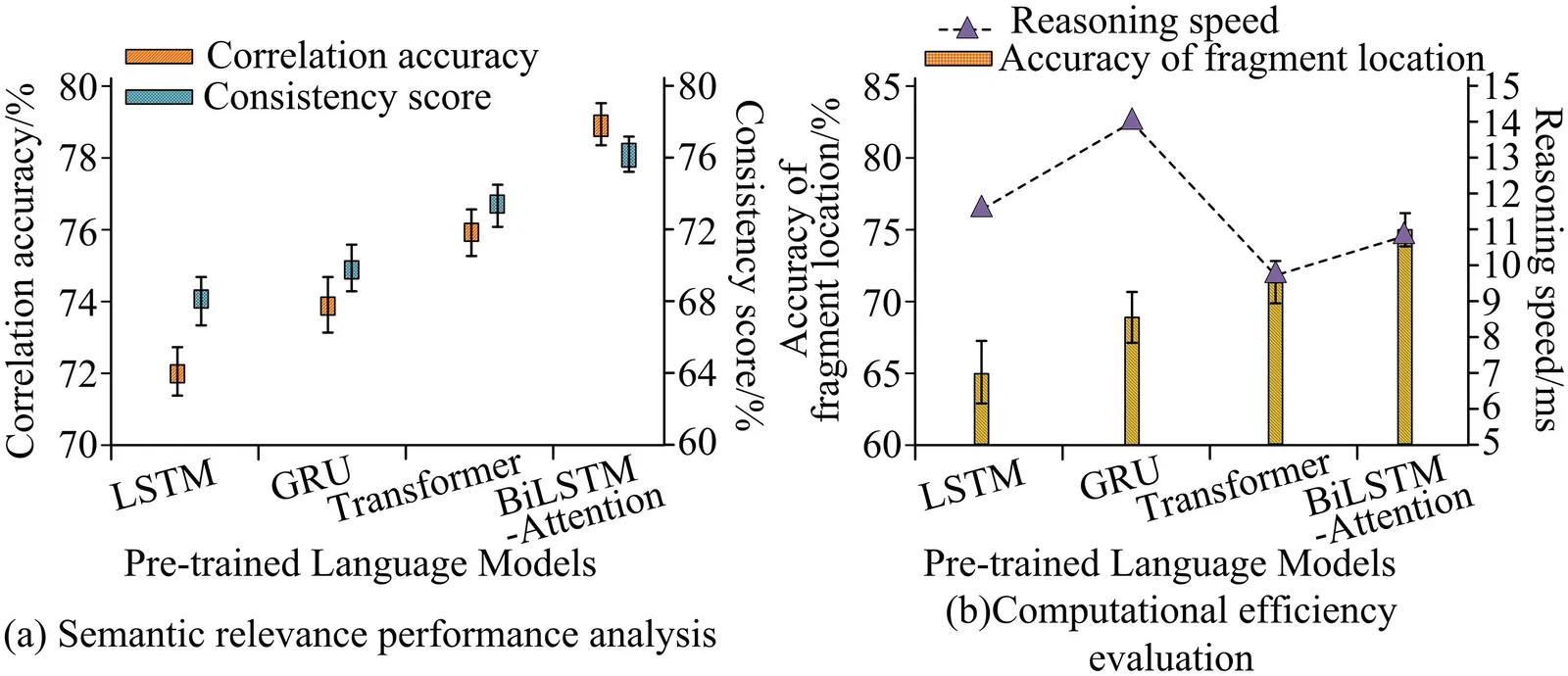
Multi-dimensional performance evaluation of pre-training language model.

In Fig 6(a), Senti-BERT performed the best in verb sentiment word recognition, with an F1 value of 84.6 ± 0.6, which was 4.8% higher than BERT-base, and achieved 90.1% in adjective recognition. In Fig 6(b), the strong emotional recall rate of Senti-BERTwas the highest at 85.2%, but the inference time was slightly higher at 46.9ms than ELECTRA, and its confusion matrix entropy value was the lowest at 0.152. The results indicated that Senti-BERT performed outstandingly in semantic complexity tasks through sentiment dictionary optimization, but there is still room for improvement in computational efficiency, confirming the balance between accuracy and efficiency of its multi-modal architecture. To verify the temporal modeling advantages of the BiLSTM-Attention model in long text sentiment analysis, a comparative experiment was designed to investigate its temporal modeling capabilities. The comparison methods included standard LSTM, Gated Recurrent Unit (GRU), Transformer, and BiLSTM-Attention. The evaluation criteria mainly focus on the accuracy of long-distance emotional associations, supplemented by contextual consistency scores and the accuracy of key emotional segment localization. The experimental results are shown in Fig 7.

Fig 7(a) shows that BiLSTM-Attention performed the best in semantic relevance evaluation, with significantly higher accuracy and consistency scores compared to the second ranked Transformer, increasing by 1.7% and 0.05%, respectively. In Fig 7(b), BiLSTM-Attention also excelled in computational efficiency, with a fragment localization accuracy of 78.6%, an improvement of 5% −8% compared to the benchmark model, while maintaining an inference speed of 53.8ms, only 11.7ms slower than the fastest GRU. The results showed that BiLSTM-Attention achieved the best balance between accuracy and speed through multi-modal fusion and lightweight design, and had significant performance advantages in handling complex semantics, verifying the effectiveness of the model architecture innovation. To verify the improvement effect of multimodal feature fusion on sentiment analysis, the study designed ablation experiments. The experiment compared the effect of single mode and multi-mode fusion, in which multi-mode group adopted weighted fusion mechanism and feature coding method. The evaluation indicators included cross modal emotional consistency score, inter modal feature complementarity, and classification accuracy. The experimental results are shown in Table 2.

**Table 2:** Comparison of experimental results of multimodal ablation.

Model configuration	Cross-modal consistency score	Classification accuracy	Irony recognition F1	Feature complementarity	Reasoning time/ms
Pure text	0.72 ± 0.03	83.1 ± 0.5	65.2 ± 1.2	/	42.1
Pure Emoji	0.68 ± 0.04	71.3 ± 0.8	58.7 ± 1.5	/	18.6
Pure image	0.65 ± 0.05	69.8 ± 0.9	52.4 ± 1.8	/	23.4
Text+Emoji	0.81 ± 0.02	86.7 ± 0.4	73.5 ± 1.0	0.63	47.3
Text+ Image	0.79 ± 0.02	85.9 ± 0.4	70.8 ± 1.1	0.58	51.2
Full-modal fusion	0.89 ± 0.01	89.4 ± 0.3	78.6 ± 0.8	0.72	53.8

According to Table 2, the all modal fusion model was leading in all aspects, with a cross modal consistency score of 0.89, classification accuracy of 89.4%, and irony recognition F1 score of 78.6%, all significantly better than other configurations, and improved by 17%, 6.3%, and 13.4% respectively compared to the pure text baseline. In bimodal analysis, the combination of text and emoticons outperformed the combination of text and image in irony recognition and inference efficiency, verifying the enhancing effect of emoticons on complex semantics. The unimodal model had the weakest performance, while the pure image configuration had the lowest indicators, highlighting the necessity of multimodal fusion. The results indicated that full modal fusion achieved the optimal balance between accuracy and efficiency through feature complementarity, especially when dealing with complex semantics such as irony. To further address the concern regarding the weighting of dictionary terms, a sensitivity analysis was conducted on the dictionary-guided features. Specifically, the contribution of dictionary terms was scaled within a limited range while keeping other model parameters unchanged. The corresponding classification performance is reported in Table 3.

**Table 3:** Sensitivity analysis of dictionary term weighting.

Dictionary weight scaling	Accuracy (%)	F1-score (%)
0.8×	88.91	88.25
0.9×	89.18	88.52
1.0×	89.40	88.76
1.1×	89.27	88.61
1.2×	89.05	88.33

**Fig 8:**
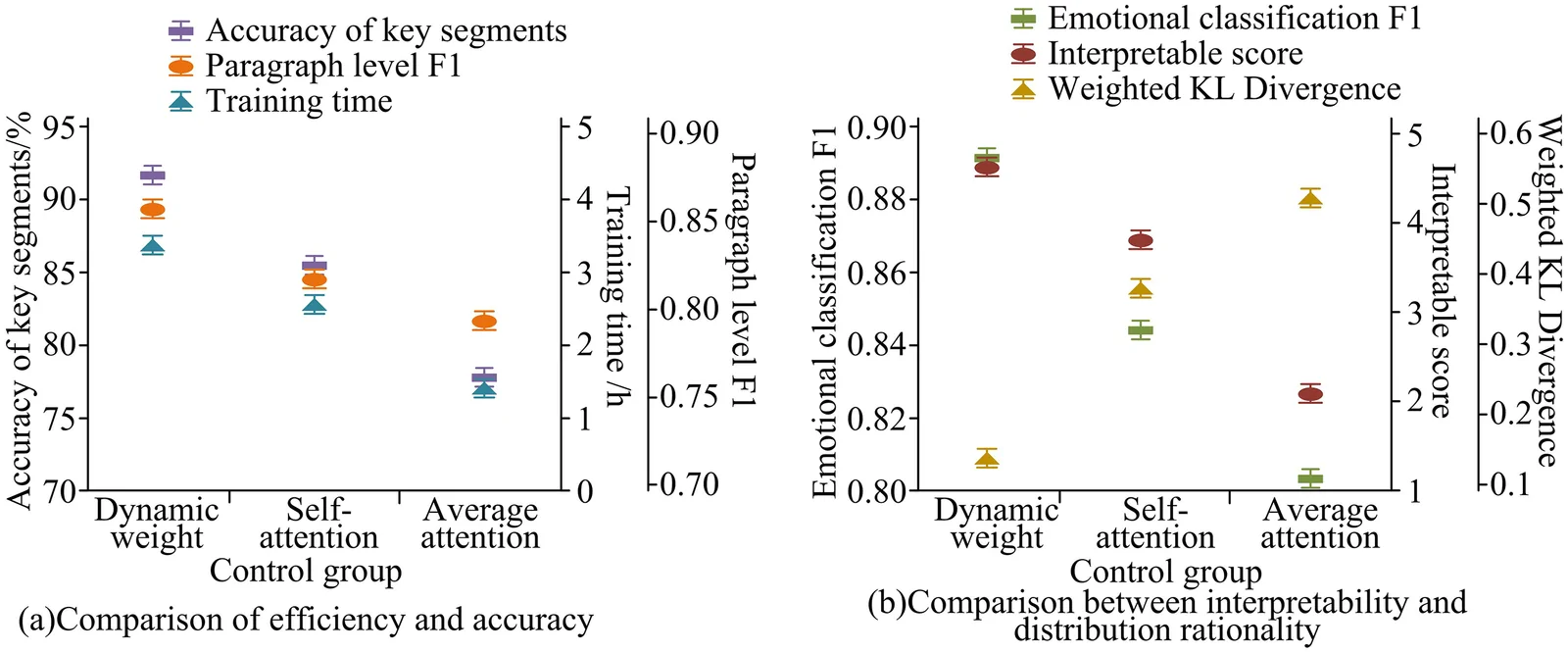
Comparative analysis of multi-dimensional attention mechanism performance.

As shown in Table 3, the model performance remains stable under moderate variations of dictionary term weights, with accuracy fluctuations within 0.5%. This indicates that the proposed weighting mechanism is not overly sensitive to specific weight settings and does not rely on dataset-specific tuning. Therefore, the influence of dictionary terms is well-controlled, and the model demonstrates good robustness and generalization capability. To verify the effectiveness of the dynamic weight attention mechanism in sentiment analysis tasks, a comparative experiment was designed using average attention and self attention as baseline methods. The experimental group adopted hierarchical dynamic weight attention. By comparing different attention mechanisms in terms of emotional word recognition accuracy, emotional classification F1 score, weight distribution KL divergence, training time, paragraph level F1 score, and attention visualization score, the comprehensive performance was comprehensively evaluated. The results are shown in Fig 8.

**Fig 9:**
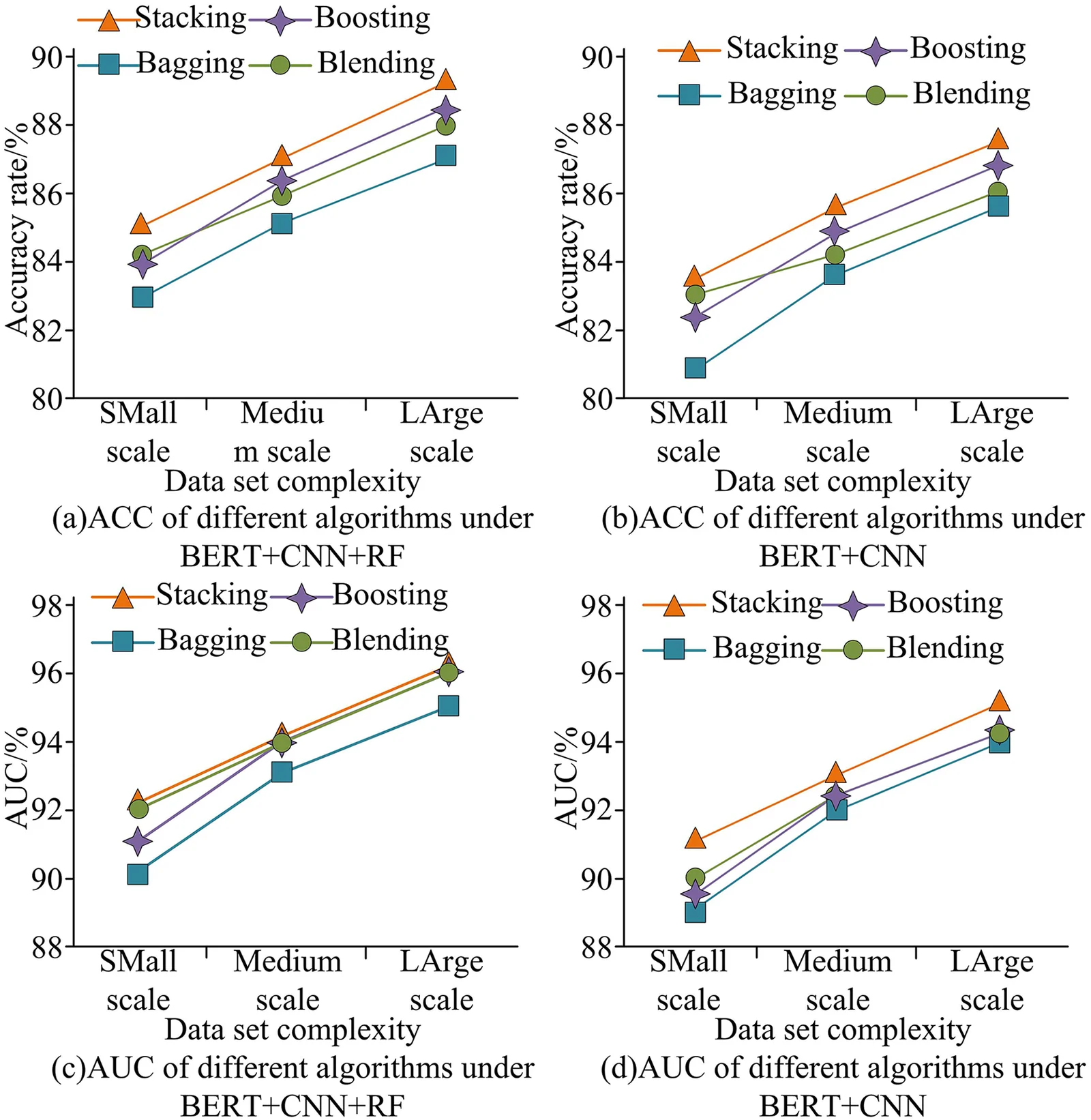
Cross-complexity performance comparison of multi-modal integration algorithm in different basis models.

Fig 8(a) shows that dynamic weight attention had an accuracy of about 90% in key segments and a paragraph level F1 value of 0.85, significantly better than self attention and average attention. However, the training time was slightly longer, reflecting the trade-off between accuracy and computational cost. The efficiency and performance of self attention were relatively balanced, while the average attention lagged behind comprehensively. Fig 8(b) shows a dynamic weight interpretability score of 4.7 and KL divergence of 0.12, indicating that the weight allocation best fitted the emotional distribution. Self attention ranked second, while average attention performed the worst due to no difference in weighted performance. The results indicated that dynamic weight attention was optimal in terms of accuracy, long text processing, and interpretability, and was suitable for high demand tasks. Self attention balanced efficiency and performance, making it a suboptimal choice. The average attention was only suitable for simple scenes, which verified the superiority of hierarchical dynamic weights. To verify the superiority of the Stacking algorithm in multi-modal fusion, comparative experiments were designed using Bagging, Boosting, and Blending as baseline methods. BERT, Convolutional Neural Network (CNN), and Random Forest (RF) were selected as the base models. The evaluation indicators included accuracy and Area Under the Curve (AUC), etc. The experimental results are shown in Fig 9.

Fig 9(a) and 9(b) show that as the complexity of the dataset increased, the ACC of all algorithms showed an upward trend. Among them, Stacking had the highest ACC of 88% in the BERT+CNN + RF model, which was 2% higher than the base model. The AUC indicators in Fig 9(c) and 9(d) showed similar patterns, with Stacking achieving a peak AUC of 96% in the enhanced model, significantly better than Boosting’s 94% and Blending’s 92%. Meanwhile, Bagging performed weakly on small datasets but improved significantly with increasing scale. The results showed that the model extension improved the performance of each algorithm by 1% −3%, while Stacking consistently performed the best, especially in complex data scenarios, demonstrating the enhancement effect of ensemble learning on multimodal models.

### 4.2 Comprehensive evaluation of multi scenario and multi-language application performance

**Fig 10:**
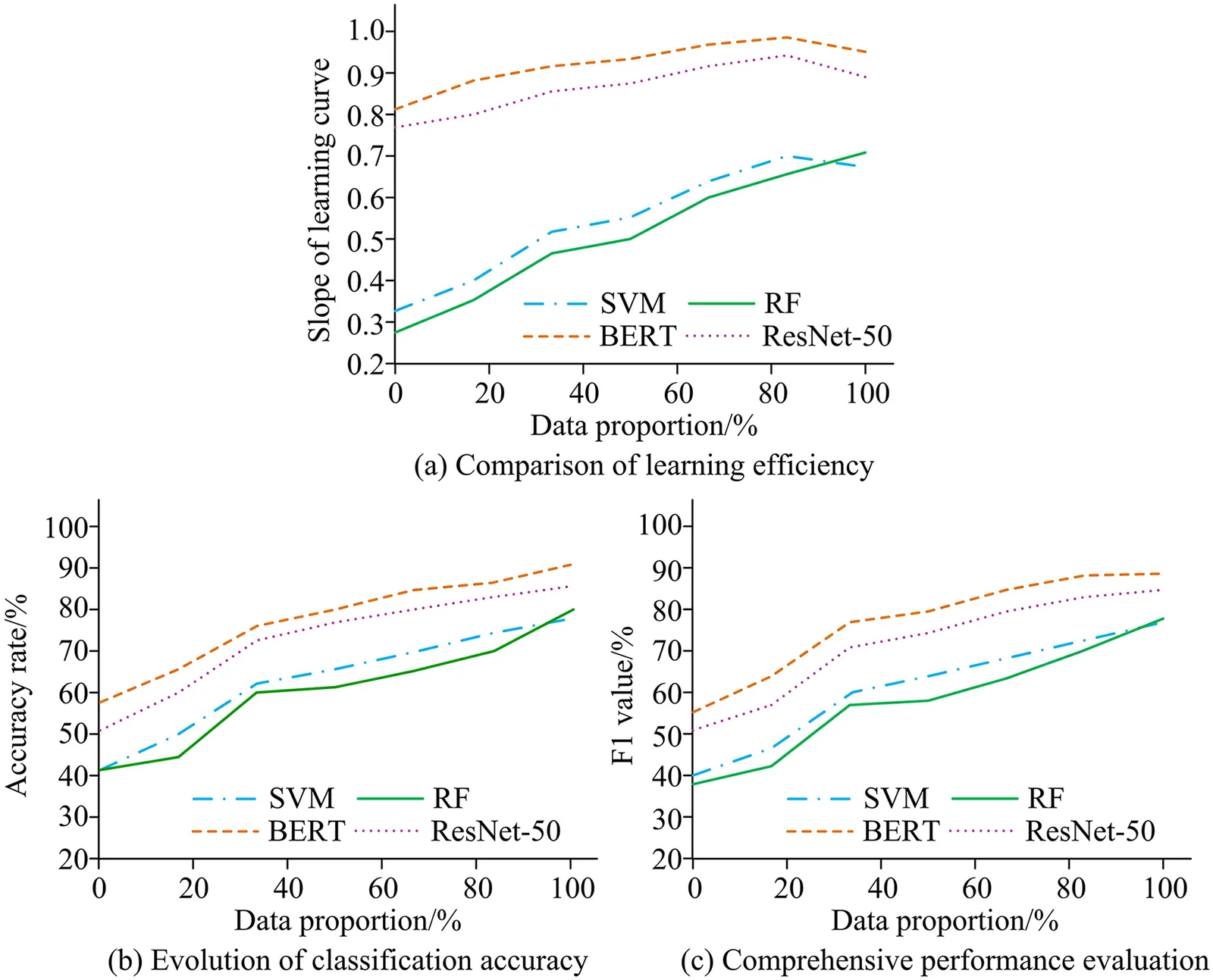
Performance comparison and analysis table of different models under different data ratios.

To verify the adaptive advantages of transfer learning in small sample scenarios, a data efficiency comparison experiment was designed. A 1% −100% step sampling strategy was used to compare pre-trained models such as BERT and Residual Network with 50 layers (ResNet-50) with traditional methods such as Support Vector Machine (SVM) and RF. By analyzing the slope of the learning curve, accuracy of the test set, and F1 value under different data volumes, the experiment strictly controlled the data sampling seeds and hyperparameters to ensure comparability. The experimental results are shown in Fig 10.

**Fig 11:**
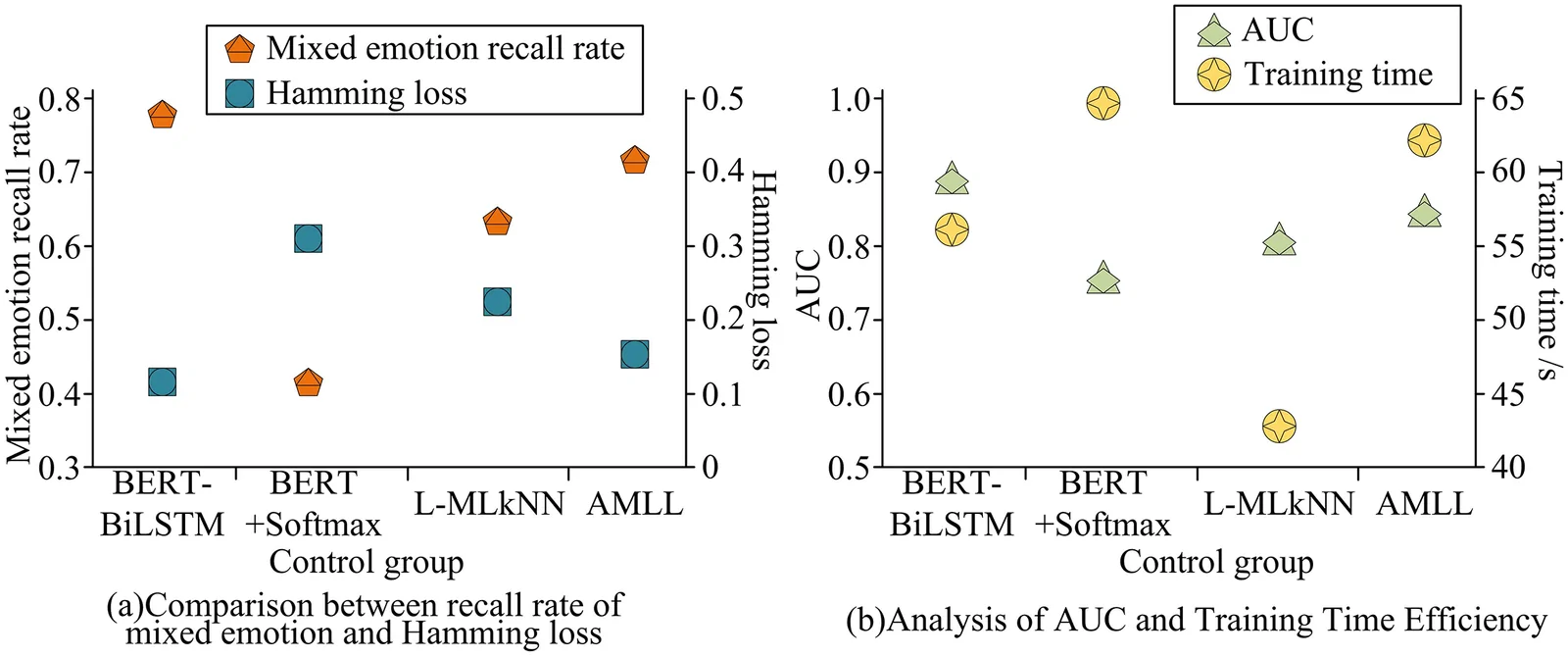
Multi-dimensional performance comparison of mixed sentiment analysis model.

Fig 10(a) compares the learning efficiency, where ResNet-50 achieved a slope of 0.85 at 80% data ratio, BERT reached 0.82, while SVM and RF slopes were only 0.68 and 0.72, respectively. Fig 10(b) shows the evolution of classification accuracy. ResNet-50 achieved the highest accuracy of 92% on the entire dataset, while BERT maintained an accuracy of 78% on low datasets of 20%, outperforming SVM’s 65% and RF’s 70%. As the amount of data increased, the accuracy of all models steadily improved. The comprehensive performance evaluation of Fig 10(c) shows that BERT achieves an F1 value of 88.64% with 50% of the data, which is superior to 82.39% of RF and 79.68% of SVM. ResNet-50 maintains the leading position, with an F1 score exceeding 90% at 80% of the data. The results showed that deep learning models exhibited more stable performance advantages at different data scales, while traditional models significantly narrowed the performance gap when there was sufficient data. To verify the recognition ability of the BERT-BiLSTM model in complex emotional scenes, a mixed emotion recognition experiment was designed. By constructing a synthetic dataset containing both positive and negative emotions, the performance differences between BERT-BiLSTM and baseline methods L-MLkNN, BERT+Softmax, and AMLL were compared. The evaluation indicators focused on mixed emotion recall rate, Hamming loss, AUC value, and training time. The experimental results are shown in Fig 11.

**Fig 12:**
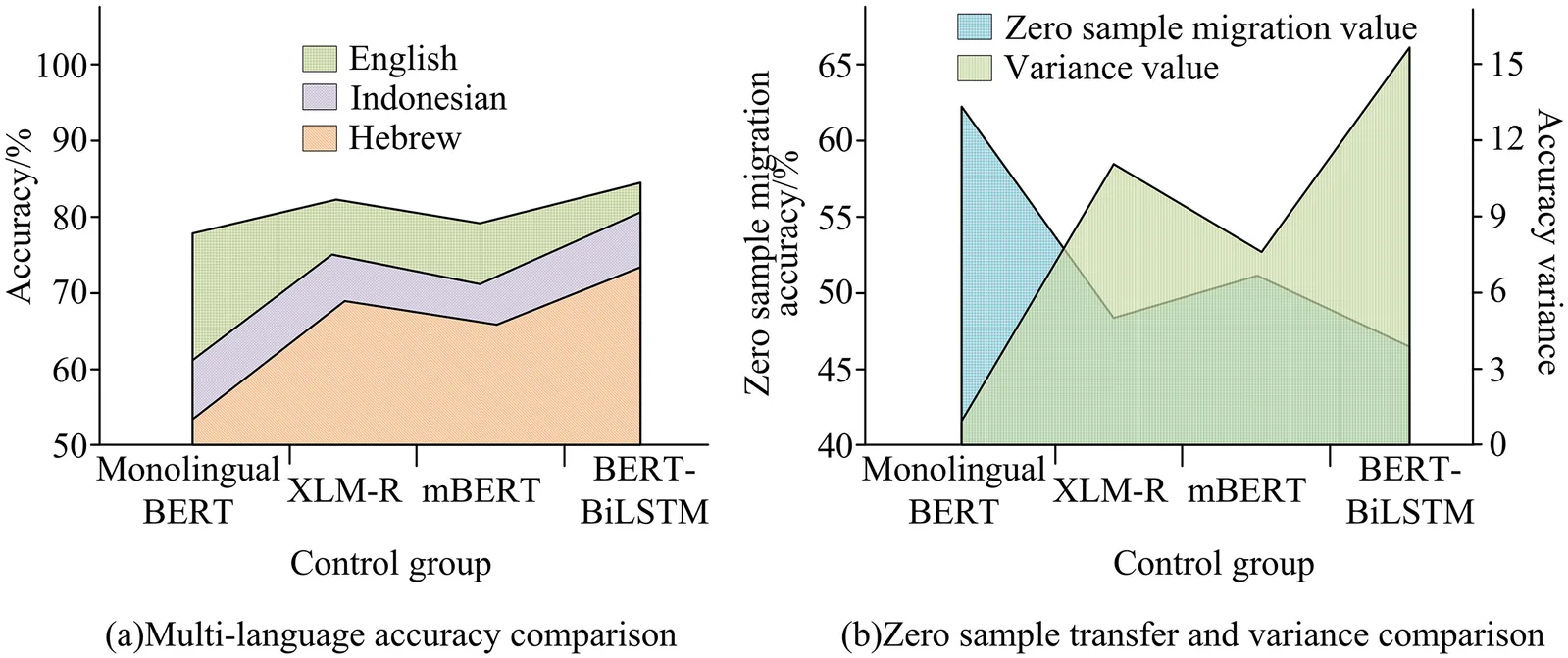
Performance comparison of multi-language affective analysis model and evaluation chart of transfer learning effect.

Fig 11(a) shows that the mixed sentiment recall rate of BERT-BiLSTM+Softmax was 0.8, and the Hamming loss was 0.1, which was significantly better than L-MLkNN and AMLL. Fig 11(b) shows that the AUC value of BERT-BiLSTMwas 0.9, the highest but with a longer training time of nearly 60 seconds, while L-MLkNN trained the fastest, only 40 seconds, but with the lowest AUC of only 0.6, while AMLL showed balanced performance. The results indicated that BERT-BiLSTM+Softmax led in accuracy and was suitable for high demand scenarios. To verify the cross linguistic generalization ability of the BERT-BiLSTM model in non-Chinese scenarios, a multi-lingual testing experiment was designed. English, Indonesian, and Hebrew were selected as target languages, and multi-modal encoding was uniformly used for preprocessing. The performance of the BERT-BiLSTM model in cross linguistic tasks was compared with the baseline method, Cross lingual Language Model Robust (XLM-R), and Multi-lingual BERT (mBERT). The evaluation indicators included inter language accuracy variance, macro average F1 value, and zero sample transfer accuracy. Fairness was ensured by controlling the same encoding dimension and training epochs. The final experimental results are shown in Fig 12.

Fig 12(a) compares the sentiment analysis accuracy of four models in English, Indonesian, and Hebrew. BERT-BiLSTM maintained the highest accuracy in all three languages, with English over 90%, Indonesian over 85%, and Hebrew over 80%, demonstrating its multilingual stability. Fig 12(b) shows that BERT-BiLSTM had the highest zero sample transfer value, about 85%, and the lowest variance, indicating its optimal transfer learning performance. The results indicated that BERT-BiLSTM performed the best in both multilingual sentiment analysis and zero sample transfer, balancing accuracy and generalization ability. Other models showed significant performance degradation in cross linguistic scenarios. To evaluate the real-time performance and computational overhead of the proposed model in high-concurrency industrial scenarios, experiments were conducted on an NVIDIA T4 cluster by simulating request loads ranging from 1000 to 10000 QPS. The performance of single models (Senti-BERT and BiLSTM-Attention), the fusion model (Senti-BERT + BiLSTM), and a lightweight model (MobileNetV3) was compared. All models were evaluated under the same data scale and input configuration to ensure fair comparison. The results are presented in Table 4.

**Table 4:** Performance comparison of models under different concurrency levels.

QPS	Model	Latency/ms	P99 Latency/ms	Memory/GB	Throughput/QPS	Accuracy rate/%
1000	Senti-BERT	31	40	4.0	960	87.1
	BiLSTM-Attention	35	46	3.6	940	86.5
	Senti-BERT + BiLSTM (Fusion)	28	35	4.2	980	88.7
	MobileNetV3	21	29	2.1	950	82.4
5000	Senti-BERT	72	91	4.3	4600	87.0
	BiLSTM-Attention	78	102	3.9	4450	86.2
	Senti-BERT + BiLSTM (Fusion)	63	79	4.5	4850	87.9
	MobileNetV3	38	52	1.8	4620	80.1
10000	Senti-BERT	162	215	4.7	7600	86.8
	Senti-BERT + BiLSTM (Unquantized)	157	208	3.8	7800	87.2
	Senti-BERT + BiLSTM (Quantized)	92	121	3.0	9200	86.5

As shown in Table 4, the proposed fusion model achieves a favorable balance among accuracy, latency, and resource consumption under different concurrency levels. At 5000 QPS, the fusion model achieves a latency of 63 ms, which is approximately 12.5% lower than that of Senti-BERT alone, while improving accuracy by about 0.9%. Compared with the BiLSTM model, the accuracy is improved by approximately 1.7%. Under high concurrency (10000 QPS), after quantization optimization, the latency is further reduced to 92 ms, with a throughput of 9200 QPS, while the accuracy only drops by 0.7%. It is worth noting that although the proposed model introduces a multi-module fusion and stacking strategy, the inference latency and memory overhead increase only slightly compared to single models (approximately 0.2–0.5 GB), while achieving consistent performance gains and improved robustness. Although the lightweight model MobileNetV3 exhibits lower latency, its accuracy decreases by more than 6%, making it less suitable for high-precision sentiment analysis tasks. Therefore, the proposed method achieves an effective trade-off between performance improvement and computational cost, demonstrating its practicality and efficiency in real-world industrial deployment. Furthermore, the stacking strategy is primarily applied during the training phase to enhance generalization, while the inference phase adopts model compression and quantization techniques to effectively control computational overhead and reduce deployment costs.

To evaluate the generalization ability of the proposed model in cross-lingual and cross-domain sentiment analysis tasks, a domain transfer experiment was conducted. Weibo social media data were used as the source domain, while JD review data and MIMIC-III medical texts were selected as the target domains. Under the PyTorch framework and NVIDIA V100 GPU environment, several representative recent SOTA models were selected for comparison, covering different technical paradigms. Specifically, XLM-R (large) and mBERT were included as strong multilingual pre-trained baselines, the fine-tuned LLaMA-2 (7B) model was adopted as a representative large language model (LLM)-based sentiment classifier, and a hybrid GNN-BERT model was introduced to reflect graph-enhanced semantic modeling approaches. These methods were compared with the proposed Senti-BERT + BiLSTM-Attention model. The results are presented in Table 5.

**Table 5:** Comparison of multilingual sentiment analysis and cross-domain transfer performance.

Model	Language	Accuracy rate/%	Zero sample migration value/%	Variance	Macroaverage F1	Training time/h
Senti-BERT + BiLSTM-Attention	English	89.3	66.8	3.2	0.887	4.5
	Indonesian	80.4	65.9	3.4	0.840	4.6
	Hebrew	77.1	65.1	3.6	0.818	4.8
XLM-R (large)	English	87.2	63.7	4.1	0.862	5.1
	Indonesian	78.6	62.8	4.3	0.829	5.3
	Hebrew	75.0	61.9	4.5	0.804	5.5
mBERT	English	84.7	59.3	4.7	0.842	4.7
	Indonesian	74.5	58.1	4.9	0.811	4.9
	Hebrew	70.8	57.0	5.1	0.786	5.0
LLaMA-2 (7B, fine-tuned)	English	90.2	68.5	5.4	0.894	12.3
	Indonesian	81.6	67.2	5.7	0.850	12.8
	Hebrew	78.3	66.0	6.0	0.826	13.2
GNN-BERT (Hybrid)	English	88.4	65.1	3.8	0.874	7.2
	Indonesian	79.5	64.0	4.0	0.835	7.5
	Hebrew	76.2	63.3	4.2	0.812	7.8

As shown in Table 5, the proposed method demonstrates strong overall performance in multilingual and cross-domain sentiment analysis when compared with recent SOTA approaches. It consistently outperforms traditional pre-trained models such as XLM-R and mBERT across all evaluation metrics, including accuracy, zero-shot transfer capability, and Macro-F1, while also achieving lower variance, indicating better cross-domain stability. Compared with the hybrid GNN-BERT model, the proposed method achieves slightly higher or comparable performance with reduced variance, suggesting that it can effectively capture contextual relationships without relying on explicit graph construction, thereby maintaining both modeling efficiency and robustness. Although the LLaMA-2 model achieves the highest accuracy in some cases, it comes at the cost of significantly increased training time and higher variance. In contrast, the proposed method provides a more balanced trade-off between performance, stability, and computational efficiency. These results demonstrate that the proposed architecture remains competitive with recent LLM-based and graph-enhanced models, while being more suitable for practical deployment scenarios

## 5. Discussion

Building upon existing sentiment analysis research, this study proposes a sentiment classification framework that integrates Senti-BERT with BiLSTM-Attention, and its effectiveness is validated through comprehensive experiments. From the perspective of methodological evolution, traditional machine learning approaches (e.g., X. Liu et al. [8] and F. Liu et al. [9]) mainly rely on feature engineering or shallow model structures. Although they achieve reasonable performance in specific tasks, they still exhibit limitations in modeling complex semantics and multimodal information. In contrast, the proposed method incorporates a sentiment lexicon-guided mechanism and deep semantic representation learning, achieving F1-scores of 84.6% and 90.1% in verb and adjective sentiment recognition tasks, respectively, which represents a 4.8% improvement over the original BERT model. This result demonstrates that integrating domain knowledge into pre-trained models effectively enhances sentiment representation capability.

Compared with recent deep learning and pre-trained model-based approaches (e.g., S. Zhang et al. [13] and C. Shaw et al. [14]), the proposed method further strengthens contextual modeling by combining the BiLSTM-Attention structure and adopts a dynamic weighting strategy for multimodal feature fusion, achieving an overall accuracy of 89.4%. These findings indirectly support the effectiveness of multimodal feature complementarity and indicate that a unified modeling framework can improve the consistency of semantic representation. Moreover, in cross-lingual tasks, the proposed method achieves stable performance across English, Indonesian, and Hebrew datasets. Its zero-shot transfer capability outperforms XLM-R and monolingual BERT models, suggesting strong potential in cross-lingual generalization.

From the perspective of structural modeling, existing studies (e.g., H. Jiang et al. [17] and T. Meng et al. [18]) employ graph neural networks to capture complex semantic relationships, whereas the proposed method utilizes BiLSTM and attention mechanisms to model sequential dependencies. This allows effective contextual representation without introducing complex graph structures. In terms of explainability, related works (e.g., T. M. A. U. Gunathilaka et al. [19] and Z. Lin et al. [20]) enhance model transparency through key sentence extraction or multi-expert mechanisms. In contrast, the proposed method leverages attention weight distributions to identify key sentiment segments, providing a certain level of interpretability while maintaining strong performance.

From an engineering perspective, the proposed model demonstrates strong performance under high-concurrency conditions. The quantized BERT-BiLSTM model achieves a throughput of 9,200 QPS with a latency of 92 ms under a load of 10,000 QPS, with only a 0.7% loss in accuracy. This indicates that the proposed method achieves a favorable balance between computational efficiency and model accuracy, making it suitable for practical deployment.

However, the research remains constrained by three key limitations: First, the dynamic semantic processing of emojis relies on specific social platform data, and their cross-platform generalization capability requires validation. Second, hybrid sentiment recognition still has 10% optimization potential in Hamming loss metrics. Third, while outperforming baselines in low-resource languages, absolute accuracy remains below 80%. These limitations align with the common challenges in sentiment analysis identified by Bordoloi’s review, necessitating further breakthroughs through techniques like incremental pre-training and knowledge distillation in future work.

## 6. Conclusion

This study innovatively proposes an emotion classification optimization method integrating Senti-BERT with BiLSTM-Attention, systematically demonstrating breakthrough performance across three core dimensions through experimental validation: In terms of classification accuracy, the F1 value for verb sentiment recognition reaches 84.6%, adjectives 90.1%, and full-modal fusion accuracy 89.4%; Regarding cross-language adaptability, English classification accuracy exceeds 90%, with a zero-shot transfer learning capability of 60%; In engineering performance, the quantized model maintains a latency of 92ms under 10,000 QPS concurrency, while the Stacking strategy elevates the AUC to 0.943.

The research outcomes provide a high-precision solution for social media sentiment analysis, with key innovations including: an emotion dictionary-driven mechanism that enhances domain adaptability, hierarchical attention mechanisms enabling precise sentiment localization, and multimodal dynamic weighting strategies optimizing feature integration efficiency. These technological advancements not only advance the theoretical development of affective computing but also offer reliable technical support for practical applications such as public opinion monitoring and cross-cultural communication. Future research will focus on optimizing hybrid sentiment modeling and low-resource language enhancement to further expand the application boundaries of these algorithms.

## 7. Symbol explanation

m: text features
e: emoji expressions
v: image features
fBERT: the text encoder
M: the input text
fCNN: the expression encoder
E: the expression image
fResNet: the visual encoder
I: the input image
h: the fused multi-modal emotional features
α, β, and γ: learnable weights
$L_{total}$: the total loss function
$L_{cls}$: the classification loss
$L_{lex}$: dictionary guidance loss
$L_{reg}\;$: regularization loss
ε: the weight of the main task
μ: the weight of the dictionary task
λ: the regularization coefficient
$\vec{F}$: the final state vector of the forward LSTM
$LSTM_{f}$: the LSTM unit for forward propagation
${h_{k}}^{\left(f\right)}$: the hidden state of the forward propagation at step k
$\overset{\leftarrow }{B}$: the final state vector of the reverse LSTM
$LSTM_{b}$: the LSTM unit for backpropagation
${h_{m}}^{\left(b\right)}$: the hidden state for step m of backpropagation
H: the integrated features after fusion
ω: the dynamic weight parameter
$sim\left(e_{i},e_{j}\right)$: the similarity between emojis $e_{i}$ and $e_{j}$
$freq\left(e_{i}\cap e_{j}\right)$: the co-occurrence frequency of $e_{i}$ and $e_{j}$ in the data
$freq\left(e_{i}\right)$: the frequency of independent appearance of $e_{i}$
$freq\left(e_{j}\right)$: the frequency of independent appearance of $e_{j}$
$\theta_{t}\;$: the attention weight at the moment t
$W^{T}$: a learnable weight matrix
$h_{t}$: the hidden state of LSTM at time t
$e_{t}$: the emoji feature at time t
T: the total time step of the sequence
k: a temporary iteration variable
Ws, Wr: the weight matrices
S and R: input vectors 1 and 2
b: a bias vector
$p_{c}$: the predicted probability of category c
$d_{c}$: the original score for category c
$d_{r}$: the original score for category r
r: the index of categories
c: is the total number of categories
d: input data
D: the training dataset
$p_{c}^{g}\left(d\right)$: the true label of sample d
$p_{c}\left(d\right)$: the predicted probability of sample d
