# Supplementary material for: Sentiment classification optimization based on improved Senti-BERT and BiLSTM-Attention
Source: PLoS One. 2026 Sep 15;21(9):e0358103. doi: 10.1371/journal.pone.0358103 (PMC13577358; doi:10.1371/journal.pone.0358103)
Supplement: Supplementary file 3 [file pone.0358103.s004.doc]

Kindly note that the provided link currently contains only duplicated materials already presented in the manuscript, such as tables and figures, which does not meet the journal’s requirements for transparency and reproducibility. The authors are required to provide the complete set of materials necessary to fully reproduce the study, including the raw data underlying the analyses, the full code used to generate the results, and clear instructions that enable replication of all tables and figures. In addition, the concerns raised in the previous review round remain insufficiently addressed.

Reply: Thank you very much for your comments. The original data and the full code for generating the results have all been provided.

The data file of the manuscript is available at: https://zenodo.org/records/20502233

The "Code" file of the manuscript is available at: https://zenodo.org/records/21275245

Specifically, the sources of all equations must be clearly identified and properly cited, with appropriate attribution to the original works in which they were first proposed or applied, and the quality of the figures must be improved to meet publication standards. Please note that this revision will be considered a final opportunity to adequately address these issues, and failure to comply may result in rejection of the manuscript.

Reply: Thank you very much for your comments. The source of the equations has been provided and the quality of the figures has been improved. As follows:

The formulation in equation (1) is based on the standard feature representation process of pretrained language models, convolutional feature extraction, and residual visual encoding. In this study, this general encoding framework is adapted to obtain unified vector representations of text, emoji, and image modalities [26].

The fusion form in equation (2) follows the common weighted multimodal fusion strategy. Different from fixed-weight concatenation, the weights in this study are learnable parameters, which allow the model to dynamically adjust the contribution of text, emoji, and image features during training [27].

The loss function in equation (3) is designed according to the multi-objective optimization idea commonly used in deep learning. The classification loss is used as the main task objective, while the dictionary-guided loss and regularization loss are introduced as auxiliary constraints to enhance sentiment representation and reduce overfitting [28].

The bidirectional modeling process in equation (4) is based on the standard BiLSTM sequence representation structure. In this study, the forward and backward hidden states are further integrated through a dynamic weighting parameter to strengthen contextual sentiment representation [29].

The similarity measure defined in equation (5) is derived from co-occurrence statistics and is conceptually related to cosine-normalized association measurement. In this study, it is adapted to evaluate the semantic association strength between emojis by normalizing their co-occurrence frequency with their independent occurrence frequencies [30].

The attention weight calculation in equation (6) is based on the general neural attention mechanism. In this study, the attention score is calculated by jointly considering the BiLSTM hidden state and emoji feature at each time step, so that key emotional segments and context-dependent emoji semantics can be assigned higher weights [31].

The nonlinear transformation in equation (7) follows the standard neural feature mapping form. It projects different input representations into a shared hidden space through learnable weight matrices and a nonlinear activation function, thereby enhancing the interaction between heterogeneous semantic features [32].

The Softmax function and cross-entropy loss in equation (8) are standard classification formulations in neural network-based sentiment classification. They are used to convert sentiment scores into category probabilities and optimize the model parameters according to the ground-truth labels [33].

[26]Y. Zhao, M. Mamat, A. Aysa, and K. Ubul, “Multimodal sentiment system and method based on CRNN-SVM,” Neural Comput. Appl., vol. 35, no. 35, pp. 24713-24725, Dec. 2023, 10.1007/s00521-023-08366-7.

[27]H. Zhang, “Multimodal sentiment analysis method based on hierarchical adaptive feature fusion network,” Int. J. Semant. Web Inf. Syst., vol. 20, no. 1, pp. 1-23, Jan. 2024, 10.4018/IJSWIS.335918.

[28]Z. A. Khan, Y. Xia, A. Khan, M. Sadiq, M. Alam, F. A. Awwad, and E. A. A. Ismail, “Developing lexicons for enhanced sentiment analysis in software engineering: An innovative multilingual approach for social media reviews,” Comput. Mater. Contin., vol. 79, no. 2, pp. 2771-2793, May 2024, 10.32604/cmc.2024.046897.

[29]M. K. Anam, M. Munawir, L. Efrizoni, N. Fadillah, W. Agustin, I. Syahputra, et al., “Improved performance of hybrid GRU-BiLSTM for detection emotion on Twitter dataset,” J. Appl. Data Sci., vol. 6, no. 1, pp. 354-365, Jan. 2025, 10.47738/jads.v6i1.459.

[30]G. Salton, A. Wong, and C. S. Yang, “A vector space model for automatic indexing,” Commun. ACM, vol. 18, no. 11, pp. 613-620, Nov. 1975, 10.1145/361219.361220.

[31]W. Li, S. Yin, and T. Pu, “Lexical attention and aspect-oriented graph convolutional networks for aspect-based sentiment analysis,” J. Intell. Fuzzy Syst., vol. 42, no. 3, pp. 1643-1654, Jan. 2022, 10.3233/JIFS-211045.

[32]Y. Zhi, J. Li, H. Wang, J. Chen, and W. Wei, “A multimodal sentiment analysis method based on fuzzy attention fusion,” IEEE Trans. Fuzzy Syst., vol. 32, no. 10, pp. 5886-5898, Oct. 2024, 10.1109/TFUZZ.2024.3434614.

[33]Z. Zhou, H. Huang, and B. Fang, “Application of weighted cross-entropy loss function in intrusion detection,” J. Comput. Commun., vol. 9, no. 11, pp. 1-21, Nov. 2021, 10.4236/jcc.2021.911001.
