# Supplementary material for: Sentiment classification optimization based on improved Senti-BERT and BiLSTM-Attention
Source: PLoS One. 2026 Sep 15;21(9):e0358103. doi: 10.1371/journal.pone.0358103 (PMC13577358; doi:10.1371/journal.pone.0358103)
Supplement: Supplementary file 4 [file pone.0358103.s005.doc]

Additional Editor Comments:

While the equations issues are met, the code issue remains. The Zenodo repository currently contains only duplicated figures and tables and does not meet the journal’s reproducibility requirements. The authors must provide the actual data used in the study, together with the complete executable code and, where appropriate, well-documented Jupyter notebooks.

Reply: Thank you for your feedback. In response to the concerns regarding reproducibility, the Zenodo repository has been updated with the complete executable source code, fixed experimental parameters, data preprocessing procedures, input data schema, sample files, and reproduction instructions. The raw Sina Weibo data cannot be redistributed because of platform authorization and user privacy restrictions. Instead of Jupyter notebooks, a standalone executable Python script with detailed comments and a complete training-evaluation workflow has been provided. Please refer to the revised “Code” document and the updated Zenodo repository.

The "Code" file of the manuscript is available at: https://zenodo.org/records/21275245
