# Supplementary material for: Sentiment classification optimization based on improved Senti-BERT and BiLSTM-Attention
Source: PLoS One. 2026 Sep 15;21(9):e0358103. doi: 10.1371/journal.pone.0358103 (PMC13577358; doi:10.1371/journal.pone.0358103)
Supplement: Supplementary file 5 [file pone.0358103.s006.doc]

PONE-D-25-48304R4

Sentiment Classification Optimization Based on Improved Senti-BERT and BiLSTM-Attention

PLOS One

The materials provided include a repository containing figure and table outputs, which are results rather than the underlying research data required for reproduction. If a separate Zenodo repository contains the complete source code and the raw data used to generate the reported results, please clearly identify this repository and ensure that it includes adequate documentation and instructions for reproducing all analyses, tables, and figures. At present, it is not possible to verify compliance with the journal's reproducibility requirements. Please treat this as a final opportunity to clarify and provide the necessary materials.

**Reply:** Thank you for your careful comment and for emphasizing the journal’s reproducibility requirements. We apologize for the confusion caused by the previously provided repository, which only contained generated figure and table outputs rather than the original source materials.

We have now deposited the complete source code, raw experimental data, and all supporting materials required to reproduce our analyses, tables and figures into a dedicated Zenodo repository: https://zenodo.org/records/22030815

This archive includes:

Full implementation code for our improved Senti‑BERT and BiLSTM‑Attention model, including data pre‑processing scripts, model training, evaluation, multimodal feature fusion, and Stacking multi‑model fusion modules;

Raw experimental datasets (text, emoji and image‑related data) used in this study;

A detailed README documentation file, covering experimental environment configuration, dependency package versions, hyper‑parameter settings, step‑by‑step running instructions, and guidance for reproducing all experimental results, tables and figures presented in the manuscript.

All scripts are configured with relative paths, so users can reproduce the reported F1‑score, classification accuracy, AUC and other experimental metrics by following the instructions in README. We have also updated the Data Availability Statement within the revised manuscript, adding this Zenodo link for permanent reference.

We believe this supplementary material now fully satisfies the journal’s reproducibility requirements.

**CODE AND DATA AVAILABILITY**

The complete source code, raw experimental data, and all supporting materials required to reproduce the analyses, tables and figures have been deposited into a dedicated Zenodo repository: <https://zenodo.org/records/22030815>
