# Supplementary material for: Sentiment classification optimization based on improved Senti-BERT and BiLSTM-Attention
Source: PLoS One. 2026 Sep 15;21(9):e0358103. doi: 10.1371/journal.pone.0358103 (PMC13577358; doi:10.1371/journal.pone.0358103)
Supplement: Supplementary file 1 [file pone.0358103.s002.docx]

Reviewer #1: Sentiment analysis is an active research area for the last 2 decades. On this paper the others are worked on improved senti-BERT and Bilstm attention model. Overall, good work, but needs major changes for improvement.

1) introduction and related work is merged into one section. It is expected that introduction should be brief and concise. To many details are given in introduction. A separate related work section is expected highlighting research gap.

Reply: Thank you very much for your comments. The Introduction section has been simplified and a separate Related Work section has been provided to highlight the research gap. The research gap is shown as follows:

In summary, although significant progress has been achieved in sentiment classification, several challenges remain. Most existing methods focus on single-modality modeling and lack effective multimodal fusion capabilities, which limits their ability to capture complementary information across heterogeneous data sources. In addition, limitations persist in modeling complex semantic relationships and long-range dependencies, especially in short texts with sparse contextual information and implicit sentiment expressions. Furthermore, many deep learning models rely heavily on large-scale parameters, leading to high computational costs and reduced efficiency in real-world deployment scenarios. Meanwhile, the issue of model interpretability remains insufficiently addressed, as most methods fail to provide transparent explanations for their predictions, thereby reducing their reliability in sensitive application domains. Moreover, cross-domain generalization ability is still weak, and models often suffer from performance degradation when applied to different datasets or languages.

2) figure 2. the orange boxes in Bi-lstm attentions or not labelled. Also four condition in figure one or also not showing their purpose

Reply: Thank you very much for your comments. All the boxes in this Figure 2 has been labelled and the purpose of the four condition in Figure 1 has been shown. As follows:

**Figure 2. BiLSTM model framework**

**Figure 1. BERT-BiLSTM model frame diagram**

As shown in Figure 1, the proposed model consists of four main modules: the data processing module, the Senti-BERT feature extraction module, the BiLSTM-Attention contextual modeling module, and the emotional classification module. First, in the data processing stage, the raw text is preprocessed by removing noise using regular expressions (e.g., HTML tags and URLs), performing Chinese word segmentation, and filtering stop words, thereby improving data quality and providing standardized input for subsequent modeling [21-22]. Then, in the Senti-BERT module, the preprocessed text is transformed into vector representations using a pre-trained language model. Meanwhile, a sentiment lexicon-guided mechanism is introduced to enhance the encoding of sentiment words and opinion words, thereby strengthening the representation of emotional semantic features. This mechanism dynamically balances semantic and sentiment information to improve sentiment recognition accuracy [23-24]. Subsequently, the BiLSTM-Attention module further models the sequential features. The bidirectional long short-term memory network captures contextual dependencies in both forward and backward directions, while the attention mechanism assigns different weights to features at different time steps, highlighting key emotional information and generating more discriminative contextual representations [25]. Finally, in the emotional classification module, the fused feature vectors are fed into a Softmax classifier to predict the sentiment categories. Through multi-level feature extraction and fusion, the proposed framework achieves an end-to-end mapping from raw text to sentiment labels.

3)fig 10 a. Also data proportion with values of 1,5, 10 percentages is not proper experimentation. For same fig, c, why not values of F1-score is not in percentages like accuracy.

Reply: Thank you very much for your comments. In Figure 10(a), the data proportions have been reset to 0, 20, 40, 60, 80, and 100 percentages. In Figure 10(c), the values of the F1 score have been changed to be presented in the form of percentages. As follows:

**Figure 10. Performance comparison and analysis table of different models under different data ratios**

The comprehensive performance evaluation of Figure 10(c) shows that BERT achieves an F1 value of 88.64% with 50% of the data, which is superior to 82.39% of RF and 79.68% of SVM. ResNet-50 maintains the leading position, with an F1 score exceeding 90% at 80% of the data.

4) table 3. Captions are written in poor way. It is better to change title of tables in improved way

Reply: Thank you very much for your comments. The title of Table 3 has been changed to "Performance comparison of models under different concurrency levels".

5) How figure 3 is created the details should be Mentioned.

Reply: Thank you very much for your comments. Details of the creatation of Figure 3

have been explained. As follows:

To capture the semantic relationships among emojis, this study constructs an emoji relationship network based on the co-occurrence characteristics of high-frequency emojis in the dataset. Specifically, the co-occurrence frequency of different emojis within the same text is first calculated, and their contextual representations are used to compute cosine similarity as a measure of semantic association strength. Subsequently, emojis are treated as nodes and similarity values as edge weights to build a topological structure, which is then visualized as the network shown in Figure 3.

6) Equation Shares the similarity Between emojis, however what is the source behind this Index. Is it Adopted from cosine similarity Or any other method Because Coefficient Jacquard coefficient and Are usually used But this formula is different than all of those three.

Reply: Thank you very much for your comments. The explanation for equation (5) has been added. As follows:

The similarity measure defined in equation (5) is derived from co-occurrence statistics and is conceptually related to cosine similarity. Specifically, it normalizes the co-occurrence frequency of emoji pairs by their individual occurrence frequencies, enabling a more balanced representation of their semantic association.

7) Table 1: summary of experimental basic settings. There is no need to add the technical detailed description In so much. Caesar paragraph

Reply: Thank you very much for your comments. Table 1 has been modified, as follows:

Table 1. Summary of experimental basic settings

| Configuration category | Parameter | Configuration |
| --- | --- | --- |
| Hardware | GPU | NVIDIA V100 / A100 / T4 |
|  | Memory | 32 GB DDR4 |
|  | Storage | NVMe SSD (1 TB) |
| Software | Python | 3.8+ |
|  | Framework | PyTorch 1.12.0 |
|  | CUDA/cuDNN | CUDA 11.7 / cuDNN 8.5 |
| Model | Senti-BERT | 12 layers, 768 hidden size, 12 heads |
|  | BiLSTM | 2 layers, 512 units |
|  | Attention | Dynamic attention (64-dim) |
| Training | Optimizer | AdamW (5e-5) |
|  | Batch size | 32 |
|  | Epochs | 50 |
|  | Loss | Composite loss (main task weight 0.7) |
| Data | Dataset | 50,000 Weibo samples |
|  | Split | 8:1:1 |
|  | Text length | 512 |
|  | Image size | 224 × 224 |
| Reproducibility | Random seed | 42 |
|  | Initialization | Xavier |
|  | Early stopping | 5 epochs |

8) In addition, related work should be improved a lot. As the references are very old. So it shows that the authors have not focused on this part. So the following research articles are recommended to be cited and more related work are explored and cited in the paper. The papers should be from top journals and published in last three years only. Few latest publications focusing on GNN, XAI etc are shared for discussion :

(2025). Fine-Grained Feature Extraction in Key Sentence Selection for Explainable Sentiment Classification Using BERT and CNN. IEEE Access.

(2025). Fpa-GCN: enhancing aspect sentiment triplet extraction with feature-rich prediction-aware graph convolutional networks. Applied Intelligence, 55(9), 740. doi: 10.1007/s10489-025-06313-8

(2024). A multi-message passing framework based on heterogeneous graphs in conversational emotion recognition. Neurocomputing, 569, 127109. doi: https://doi.org/10.1016/j.neucom.2023.127109

(2025). MLM-EOE: Automatic Depression Detection via Sentimental Annotation and Multi-Expert Ensemble. IEEE Transactions on Affective Computing, 1-18. doi: 10.1109/TAFFC.2025.3585599

Reply: Thank you very much for your comments. The suggested literature focusing on GNN have been cited to enhance the relevant work section. As follows:

In recent years, sentiment analysis has further progressed toward complex structural modeling and explainability. On one hand, Graph Neural Networks (GNNs) have been introduced to capture intricate semantic dependencies by constructing relational graphs. H. Jiang et al. proposed the Fpa-GCN model, which enhances aspect sentiment triplet extraction through multi-branch graph convolution and gating mechanisms [17]. T. Meng et al. developed a heterogeneous graph-based multi-message passing framework to effectively model multimodal interactions in conversational emotion recognition tasks [18]. On the other hand, explainable sentiment analysis has gained increasing attention. T. M. A. U. Gunathilaka et al. proposed a fine-grained feature extraction approach based on BERT and CNN, improving interpretability through key sentence selection [19]. Z. Lin et al. introduced a multi-expert ensemble model that leverages multimodal sentiment annotation and multi-scale modeling, achieving improved performance in depression detection while enhancing model interpretability and robustness [20].

[17]H. Jiang, X. Chen, D. Miao, H. Zhang, X. Qin, X. Gu, and X. Li, “Fpa-GCN: Enhancing aspect sentiment triplet extraction with feature-rich prediction-aware graph convolutional networks,” [18]Appl. Intell., vol. 55, no. 9, p. 740, 2025, doi: 10.1007/s10489-025-06313-8.

[18]T. Meng, Y. Shou, W. Ai, J. Du, H. Liu, and K. Li, “A multi-message passing framework based on heterogeneous graphs in conversational emotion recognition,” Neurocomputing, vol. 569, p. 127109, 2024, 10.1016/j.neucom.2023.127109.

[19]T. M. A. U. Gunathilaka, J. Zhang, and Y. Li, “Fine-grained feature extraction in key sentence selection for explainable sentiment classification using BERT and CNN,” IEEE Access, vol. 13, pp. 68462–68480, 2025, 10.1109/ACCESS.2025.3560482.

[20]Z. Lin, Y. Wang, Y. Zhou, F. Du, and Y. Yang, “MLM-EOE: Automatic depression detection via sentimental annotation and multi-expert ensemble,” IEEE Trans. Affect. Comput., vol. 16, no. 4, pp. 2842-2858, 2025, 10.1109/TAFFC.2025.3585599.

9) There are a number of symbols used in the manuscript. Therefore, it is recommended that a table of symbols should be added in the manuscript showing the list of all the symbols used in the manuscript along with their descriptions so that it should serve as a ready reference for the readers and it will help understandability as well.

Reply: According to your kind suggestions, a table of symbols has been added as follows:

7. SYMBOL EXPLANATION

| Symbol | Explanation |
| --- | --- |
| $m$ | text features |
| $e$ | emoji expressions |
| $v$ | image features |
| $fBERT$ | the text encoder |
| $M$ | the input text |
| $fCNN$ | the expression encoder |
| $E$ | the expression image |
| $f\mathrm{Re}sNet$ | the visual encoder |
| $I$ | the input image |
| $h$ | the fused multi-modal emotional features |
| $\alpha$, $\beta$, and $\gamma$ | learnable weights |
| $L_{total}$ | the total loss function |
| $L_{cls}$ | the classification loss |
| $L_{lex}$ | dictionary guidance loss |
| $L_{reg}\text{ }$ | regularization loss |
| $\varepsilon$ | the weight of the main task |
| $\mu$ | the weight of the dictionary task |
| $\lambda$ | the regularization coefficient |
| $\vec{F}$ | the final state vector of the forward LSTM |
| $LSTM_{f}$ | the LSTM unit for forward propagation |
| $h_{k}^{\left(f\right)}$ | the hidden state of the forward propagation at step $k$ |
| $\overleftarrow{B}$ | the final state vector of the reverse LSTM |
| $LSTM_{b}$ | the LSTM unit for backpropagation |
| $h_{m}^{\left(b\right)}$ | the hidden state for step $m$ of backpropagation |
| $H$ | the integrated features after fusion |
| $\omega$ | the dynamic weight parameter |
| $sim\left(e_{i},e_{j}\right)$ | the similarity between emojis $e_{i}$ and $e_{j}$ |
| $freq\left(e_{i}\cap e_{j}\right)$ | the co-occurrence frequency of $e_{i}$ and $e_{j}$ in the data |
| $freq\left(e_{i}\right)$ | the frequency of independent appearance of $e_{i}$ |
| $freq\left(e_{j}\right)$ | the frequency of independent appearance of $e_{j}$ |
| $\theta_{t}$ | the attention weight at the moment $t$ |
| $W^{T}$ | a learnable weight matrix |
| $h_{t}$ | the hidden state of LSTM at time $t$ |
| $e_{t}$ | the emoji feature at time $t$ |
| $T$ | the total time step of the sequence |
| $k$ | a temporary iteration variable |
| $Ws$, $Wr$ | the weight matrices |
| $S$ and $R$ | input vectors 1 and 2 |
| $b$ | a bias vector |
| $p_{c}$ | the predicted probability of category $c$ |
| $d_{c}$ | the original score for category $c$ |
| $d_{r}$ | the original score for category $r$ |
| $r$ | the index of categories |
| $c$ | is the total number of categories |
| $d$ | input data |
| $D$ | the training dataset |
| $p_{c}^{g}(d)$ | the true label of sample $d$ |
| $p_{c}(d)$ | the predicted probability of sample $d$ |

10) The results should be compared with the existing studies in the relevant literature.

Reply: Thank you very much for your comments. The comparisons with the existing studies have been added. As follows:

Building upon existing sentiment analysis research, this study proposes a sentiment classification framework that integrates Senti-BERT with BiLSTM-Attention, and its effectiveness is validated through comprehensive experiments. From the perspective of methodological evolution, traditional machine learning approaches (e.g., X. Liu et al. [8] and F. Liu et al. [9]) mainly rely on feature engineering or shallow model structures. Although they achieve reasonable performance in specific tasks, they still exhibit limitations in modeling complex semantics and multimodal information. In contrast, the proposed method incorporates a sentiment lexicon-guided mechanism and deep semantic representation learning, achieving F1-scores of 84.6% and 90.1% in verb and adjective sentiment recognition tasks, respectively, which represents a 4.8% improvement over the original BERT model. This result demonstrates that integrating domain knowledge into pre-trained models effectively enhances sentiment representation capability.

Compared with recent deep learning and pre-trained model-based approaches (e.g., S. Zhang et al. [13] and C. Shaw et al. [14]), the proposed method further strengthens contextual modeling by combining the BiLSTM-Attention structure and adopts a dynamic weighting strategy for multimodal feature fusion, achieving an overall accuracy of 89.4%. These findings indirectly support the effectiveness of multimodal feature complementarity and indicate that a unified modeling framework can improve the consistency of semantic representation. Moreover, in cross-lingual tasks, the proposed method achieves stable performance across English, Indonesian, and Hebrew datasets. Its zero-shot transfer capability outperforms XLM-R and monolingual BERT models, suggesting strong potential in cross-lingual generalization.

From the perspective of structural modeling, existing studies (e.g., H. Jiang et al. [17] and T. Meng et al. [18]) employ graph neural networks to capture complex semantic relationships, whereas the proposed method utilizes BiLSTM and attention mechanisms to model sequential dependencies. This allows effective contextual representation without introducing complex graph structures. In terms of explainability, related works (e.g., T. M. A. U. Gunathilaka et al. [19] and Z. Lin et al. [20]) enhance model transparency through key sentence extraction or multi-expert mechanisms. In contrast, the proposed method leverages attention weight distributions to identify key sentiment segments, providing a certain level of interpretability while maintaining strong performance.

From an engineering perspective, the proposed model demonstrates strong performance under high-concurrency conditions. The quantized BERT-BiLSTM model achieves a throughput of 9,200 QPS with a latency of 92 ms under a load of 10,000 QPS, with only a 0.7% loss in accuracy. This indicates that the proposed method achieves a favorable balance between computational efficiency and model accuracy, making it suitable for practical deployment.

Reviewer #2:

1-While the abstract and introduction claim to have synergy by fusing multi-modal features like text, emoticons, and images, the methodology and experiments are narrowly focused on utilizing text only. You need to explain in detail how the image features are extracted and at what layer you inject them through the dynamic weighting strategy into Senti-BERT embeddings.

Reply: Thank you very much for your comments. The process of extracting image features and injecting them into the Senti-BERT embedding in the dynamic weighting strategy have been explained as follows:

Based on the above vector representations, the image feature extraction and fusion process is further refined. Specifically, the input images are first resized to a unified scale, and a pre-trained convolutional neural network is employed to extract high-level semantic features. These visual features are then projected into the same representation space as the textual features through a linear mapping, enabling cross-modal alignment. In terms of fusion position, multimodal features are not simply concatenated at the output layer. Instead, image and emoticon features are injected after the Senti-BERT generates textual embeddings and before the deep encoding stage, allowing them to participate in subsequent semantic modeling and enhancing contextual sentiment representation. During this process, the aligned visual and emoticon features are combined with textual embeddings in an intermediate representation layer, so that they can be jointly optimized with the contextual encoding procedure rather than being treated as independent inputs. For weight allocation, a dynamic weighting strategy is adopted, where the model automatically adjusts the contribution of each modality according to its importance in different samples during training. Specifically, the model learns to assign adaptive importance to textual, visual, and emoticon features by capturing their contextual relevance within each input instance. This mechanism is implemented through a learnable gating-like structure, which enables the model to selectively emphasize informative modalities while suppressing less relevant ones. As a result, the injected image features can be dynamically enhanced when they are semantically consistent with the text, or attenuated when they introduce noise or ambiguity. This mechanism avoids the limitations of fixed weights and improves the effectiveness and robustness of multimodal fusion.

2- This is an important contribution, but the main challenge here is that there is no mathematical justification or sensitivity analysis on how much weight given to each dictionary terms. These weights should not be overfitted to a specific dataset.

Reply: Thank you very much for your comments. The design of the weight given to each dictionary term has been explained, and the sensitivity analysis has been provided. Please see details as follows:

**The design of the weight given:**

It should be noted that the multimodal fusion weights are not manually assigned but are automatically learned during training. This mechanism can be regarded as an attention-based feature selection process, which reduces the risk of overfitting to a specific dataset. Moreover, the learned weight distributions remain relatively stable across different training runs and data splits, indicating robustness to weight variations.

**The sensitivity analysis:**

To further address the concern regarding the weighting of dictionary terms, a sensitivity analysis was conducted on the dictionary-guided features. Specifically, the contribution of dictionary terms was scaled within a limited range while keeping other model parameters unchanged. The corresponding classification performance is reported in Table 3.

Table 3. Sensitivity analysis of dictionary term weighting

| Dictionary weight scaling | Accuracy (%) | F1-score (%) |
| --- | --- | --- |
| 0.8× | 88.91 | 88.25 |
| 0.9× | 89.18 | 88.52 |
| 1.0× | 89.40 | 88.76 |
| 1.1× | 89.27 | 88.61 |
| 1.2× | 89.05 | 88.33 |

As shown in Table 3, the model performance remains stable under moderate variations of dictionary term weights, with accuracy fluctuations within 0.5%. This indicates that the proposed weighting mechanism is not overly sensitive to specific weight settings and does not rely on dataset-specific tuning. Therefore, the influence of dictionary terms is well-controlled, and the model demonstrates good robustness and generalization capability.

3- The model is based on a Stacking integration of Senti-BERT and BiLSTM-Attention Provide analysis on overhead computation (Inference time, memory usage) In the case of a "multi-model stack" that is intersected with other cases, any marginal gain in accuracy needs to be weighed against added latency.

Reply: Thank you very much for your comments. Regarding the computational overhead issue caused by multi-model stacking, the analysis of indicators such as inference delay, memory usage, and throughput has been supplemented. As follows:

To evaluate the real-time performance and computational overhead of the proposed model in high-concurrency industrial scenarios, experiments were conducted on an NVIDIA T4 cluster by simulating request loads ranging from 1000 to 10000 QPS. The performance of single models (Senti-BERT and BiLSTM-Attention), the fusion model (Senti-BERT + BiLSTM), and a lightweight model (MobileNetV3) was compared. All models were evaluated under the same data scale and input configuration to ensure fair comparison. The results are presented in Table 4.

Table 4. Performance comparison of models under different concurrency levels

| QPS | Model | Latency /ms | P99 Latency /ms | Memory /GB | Throughput /QPS | Accuracy rate/% |
| --- | --- | --- | --- | --- | --- | --- |
| 1000 | Senti-BERT | 31 | 40 | 4.0 | 960 | 87.1 |
|  | BiLSTM-Attention | 35 | 46 | 3.6 | 940 | 86.5 |
|  | Senti-BERT + BiLSTM (Fusion) | 28 | 35 | 4.2 | 980 | 88.7 |
|  | MobileNetV3 | 21 | 29 | 2.1 | 950 | 82.4 |
| 5000 | Senti-BERT | 72 | 91 | 4.3 | 4600 | 87.0 |
|  | BiLSTM-Attention | 78 | 102 | 3.9 | 4450 | 86.2 |
|  | Senti-BERT + BiLSTM (Fusion) | 63 | 79 | 4.5 | 4850 | 87.9 |
|  | MobileNetV3 | 38 | 52 | 1.8 | 4620 | 80.1 |
| 10000 | Senti-BERT | 162 | 215 | 4.7 | 7600 | 86.8 |
|  | Senti-BERT + BiLSTM (Unquantized) | 157 | 208 | 3.8 | 7800 | 87.2 |
|  | Senti-BERT + BiLSTM (Quantized) | 92 | 121 | 3.0 | 9200 | 86.5 |

As shown in Table 4, the proposed fusion model achieves a favorable balance among accuracy, latency, and resource consumption under different concurrency levels. At 5000 QPS, the fusion model achieves a latency of 63 ms, which is approximately 12.5% lower than that of Senti-BERT alone, while improving accuracy by about 0.9%. Compared with the BiLSTM model, the accuracy is improved by approximately 1.7%. Under high concurrency (10000 QPS), after quantization optimization, the latency is further reduced to 92 ms, with a throughput of 9200 QPS, while the accuracy only drops by 0.7%. It is worth noting that although the proposed model introduces a multi-module fusion and stacking strategy, the inference latency and memory overhead increase only slightly compared to single models (approximately 0.2-0.5 GB), while achieving consistent performance gains and improved robustness. Although the lightweight model MobileNetV3 exhibits lower latency, its accuracy decreases by more than 6%, making it less suitable for high-precision sentiment analysis tasks. Therefore, the proposed method achieves an effective trade-off between performance improvement and computational cost, demonstrating its practicality and efficiency in real-world industrial deployment. Furthermore, the stacking strategy is primarily applied during the training phase to enhance generalization, while the inference phase adopts model compression and quantization techniques to effectively control computational overhead and reduce deployment costs.

4-The current comparison does not include recent state-of-the-art (SOTA) benchmarks as of 2023–2024. Validate that your proposed architecture is still competitive by comparing your results other, more recent LLM based sentiment classifiers or hybrid GNN-BERT models

Reply: Thank you very much for your comments. Recent LLM based sentiment classifiers (LLaMA-2) and hybrid GNN-BERT models have been applied to the comparision. As follows:

To evaluate the generalization ability of the proposed model in cross-lingual and cross-domain sentiment analysis tasks, a domain transfer experiment was conducted. Weibo social media data were used as the source domain, while JD review data and MIMIC-III medical texts were selected as the target domains. Under the PyTorch framework and NVIDIA V100 GPU environment, several representative recent SOTA models were selected for comparison, covering different technical paradigms. Specifically, XLM-R (large) and mBERT were included as strong multilingual pre-trained baselines, the fine-tuned LLaMA-2 (7B) model was adopted as a representative large language model (LLM)-based sentiment classifier, and a hybrid GNN-BERT model was introduced to reflect graph-enhanced semantic modeling approaches. These methods were compared with the proposed Senti-BERT + BiLSTM-Attention model. The results are presented in Table 5..

Table 5. Comparison of multilingual sentiment analysis and cross-domain transfer performance

| Model | Language | Accuracy rate/% | Zero sample migration value/% | Variance | Macroaverage F1 | Training time/h |
| --- | --- | --- | --- | --- | --- | --- |
| Senti-BERT + BiLSTM-Attention | English | 89.3 | 66.8 | 3.2 | 0.887 | 4.5 |
|  | Indonesian | 80.4 | 65.9 | 3.4 | 0.840 | 4.6 |
|  | Hebrew | 77.1 | 65.1 | 3.6 | 0.818 | 4.8 |
| XLM-R (large) | English | 87.2 | 63.7 | 4.1 | 0.862 | 5.1 |
|  | Indonesian | 78.6 | 62.8 | 4.3 | 0.829 | 5.3 |
|  | Hebrew | 75.0 | 61.9 | 4.5 | 0.804 | 5.5 |
| mBERT | English | 84.7 | 59.3 | 4.7 | 0.842 | 4.7 |
|  | Indonesian | 74.5 | 58.1 | 4.9 | 0.811 | 4.9 |
|  | Hebrew | 70.8 | 57.0 | 5.1 | 0.786 | 5.0 |
| LLaMA-2 (7B, fine-tuned) | English | 90.2 | 68.5 | 5.4 | 0.894 | 12.3 |
|  | Indonesian | 81.6 | 67.2 | 5.7 | 0.850 | 12.8 |
|  | Hebrew | 78.3 | 66.0 | 6.0 | 0.826 | 13.2 |
| GNN-BERT (Hybrid) | English | 88.4 | 65.1 | 3.8 | 0.874 | 7.2 |
|  | Indonesian | 79.5 | 64.0 | 4.0 | 0.835 | 7.5 |
|  | Hebrew | 76.2 | 63.3 | 4.2 | 0.812 | 7.8 |

As shown in Table 5, the proposed method demonstrates strong overall performance in multilingual and cross-domain sentiment analysis when compared with recent SOTA approaches. It consistently outperforms traditional pre-trained models such as XLM-R and mBERT across all evaluation metrics, including accuracy, zero-shot transfer capability, and Macro-F1, while also achieving lower variance, indicating better cross-domain stability. Compared with the hybrid GNN-BERT model, the proposed method achieves slightly higher or comparable performance with reduced variance, suggesting that it can effectively capture contextual relationships without relying on explicit graph construction, thereby maintaining both modeling efficiency and robustness. Although the LLaMA-2 model achieves the highest accuracy in some cases, it comes at the cost of significantly increased training time and higher variance. In contrast, the proposed method provides a more balanced trade-off between performance, stability, and computational efficiency. These results demonstrate that the proposed architecture remains competitive with recent LLM-based and graph-enhanced models, while being more suitable for practical deployment scenarios.

5- The paper contains a hierarchical attention mechanism on top of the BiLSTM. A note on whether this is referring to WORD-LEVEL and SENTENCE-LEVEL attention or some other hierarchy would also be helpful, as the current description is too vague for reproducibility.

Reply: Thank you very much for your comments. The hierarchical attention mechanism adopted in this study does not follow the conventional word-level and sentence-level structure. Instead, it consists of a time-step attention over BiLSTM outputs and a multimodal attention layer for integrating textual, emoticon, and image features, enabling multi-level sentiment representation.

6- The document indicates that you are using "stacking method" for integration but is insufficient in describing the meta-learner. Be explicit about whether it is a simple logistic regression, neural network or other classifier and explain why this particular meta-learner was selected as opposed to simple averaging or voting.

Reply: Thank you very much for your comments. The meta-learner used in the stacking fusion stage has been clarified as follows:

In this study, logistic regression is adopted as the meta-learner to perform weighted fusion of the outputs from base models. This selection is grounded in the observation that the base learners (e.g., Senti-BERT and BiLSTM-Attention) already generate discriminative probabilistic predictions, such that the primary objective of the stacking stage is to recalibrate and integrate these outputs rather than to introduce additional nonlinear feature transformations. Logistic regression operates directly in the probability space, ensuring consistency in probabilistic interpretation while enabling effective aggregation of heterogeneous model outputs. Furthermore, considering that the meta-level training data in stacking are typically limited, the use of a low-capacity linear model helps mitigate the risk of overfitting and enhances generalization stability. Compared with simple averaging or voting strategies, logistic regression can adaptively learn the contribution of each base model based on its predictive reliability, thereby providing a more principled and flexible fusion mechanism.

7- A better breakdown of the data sets used, specifically class distribution (positive vs negative vs neutral) If datasets have an imbalanced target, it is better to report the F1-scores or Area Under the Precision-Recall Curve (AUPRC) rather than accuracy alone to show a model's stability. ts)

Reply: Thank you very much for your comments. A statistical analysis of the dataset was conducted, showing that the proportions of positive, negative, and neutral samples are approximately 35%, 33%, and 32%, respectively.
